# Comparative evaluation of filtration techniques for ECG signal denoising with emphasis on stationary wavelet transform

**DOI:** 10.1038/s41598-025-26476-1

**Published:** 2025-11-27

**Authors:** Norbert Ádám, Dávid Val’ko, Zoltán Balogh, Branislav Madoš, Ján Hurtuk

**Affiliations:** 1 Department of Computing and Informatics, Faculty of Electrical Engineering and Informatics, Technical University of Košice, Košice, 042 00 Slovakia; 2 Department of Informatics, Faculty of Natural Sciences and Informatics, Constantine the Philosopher University, Nitra, 949 01 Slovakia; 3 Kandó Kálmán Faculty of Electrical Engineering, Óbuda University, Budapest, Hungary

**Keywords:** Electrical and electronic engineering, Data processing

## Abstract

This paper explores filtration techniques for processing ECG signals, focusing on the evaluation of effective denoising methods. We highlight the effectiveness of Stationary Wavelet Transform as the most suitable approach for denoising ECG signals while preserving critical cardiac features. Stationary Wavelet Transform’s superior performance was validated through rigorous testing, making it a very good choice for ECG signal filtration. We have also investigated other filtration techniques, including high-pass, Chebyshev type II, Kalman, notch, Savitzky-Golay Smoothing, Moving Average, Empirical Mode Decomposition, Empirical Wavelet Transform, and Stationary Wavelet Transform filtering (with tested wavelets ’db4’, ’db5’, ’db6’, ’sym4’, ’sym5’, ’coif3’, ’coif4’, ’coif5’, ’bior3.5’, and ’rbio3.9’), to assess their effectiveness in ECG signal processing. The paper highlights the advantages of the Stationary Wavelet Transform filtration technique in maintaining signal integrity for subsequent analysis. The optimal configuration, determined to be the wavelet type ‘rbio3.9’ at level 5 with a threshold scale of 0.5, balances effective noise reduction with the preservation of crucial ECG signal details, thereby significantly contributing to clinical ECG analysis and advancing diagnostic precision.

## Introduction

Cardiovascular diseases are the main causes of death among humans globally. The early detection, diagnosis, and treatment of cardiovascular illness, together with the delivery of properly selected treatment, would contribute to reducing the current mortality rate. Diseases of the cardiovascular system can take many forms. One of those forms is an irregular heartbeat, which can be a symptom of cardiac rhythm abnormalities and is often unnoticeable by the patient. These abnormalities can interfere with the heart’s ability to pump blood effectively, leading to serious health risks. However, medical professionals have access to a variety of tools that can assist them in diagnosing pathologies in patients. These tools record a large amount of patient data, which must be evaluated by medical professionals. These data need to be properly processed before any professional can have a look at them and diagnose the patient. In this paper, we will be talking about choosing a proper filtration method that does not diminish electrocardiogram (ECG) characteristics and maintains important features of ECG. ECG signals are vital diagnostic tools widely employed to monitor cardiac health and detect heart-related abnormalities. However, raw ECG recordings frequently suffer from various forms of noise and interference, including baseline wander, power-line interference (PLI), muscle artifacts, and electrode movement artifacts. The presence of such noise can severely compromise the accuracy and reliability of subsequent analyses, potentially leading to misdiagnosis or the overlooking of critical cardiac events.

Efficient denoising methods are thus essential to enhance signal quality while preserving vital morphological features required for accurate interpretation and diagnosis. Although numerous filtering techniques exist, such as high-pass filtering, notch filtering, Savitzky-Golay Smoothing (SGS) filtering, Moving Average (MA) filtering, Kalman and the Stationary Wavelet Transform (SWT), the relative effectiveness of these methods in balancing noise removal against feature preservation remains uncertain. Given the crucial importance of ECG data integrity, this research aims to comparatively evaluate these prominent denoising techniques. The central hypothesis guiding this investigation is: “Which filtration technique among various denoising methods is most effective in preserving critical signal features while minimizing noise, thereby improving the quality of ECG data for subsequent analysis with computational demands sufficiently low to enable its integration into wearable devices intended for continuous patient monitoring?” By systematically analyzing the strengths and limitations of each filtering approach, this study seeks to identify the most suitable method, thus enabling improved diagnostic accuracy, enhanced clinical decision-making, and better patient outcomes. The primary contribution of this paper includes a comprehensive comparative evaluation of several prominent ECG denoising techniques, specifically high-pass filtering, Chebyshev type II, Kalman filtering, MA filtering, notch filtering, SGS filtering, Empirical Mode Decomposition (EMD) filtering, Empirical Wavelet Transform (EWT) filtering, and SWT filtering. Through extensive quantitative analysis based on performance metrics such as Mean Squared Error (MSE), Root Mean Square Error (RMSE), Peak Signal-to-Noise Ratio (PSNR), Percentage Root-Mean-Square Difference (PRD), Correlation Coefficient (Pearson’s r), Signal-to-Noise Ratio (SNR), kurtosis, skewness, pre-normalized standard deviation, Interquartile Range (IQR), and the preservation of clinically relevant morphological features, this study provides clear insights into the efficacy of each method. Additionally, the paper identifies the optimal filtering strategy that balances effective noise reduction with the preservation of crucial ECG signal details, thereby significantly contributing to clinical ECG analysis and advancing diagnostic precision.

## Related works

This paper examines a variety of filtration techniques for the processing of ECG signals, with a particular emphasis on the SWT as a highly effective method for denoising while preserving necessary cardiac features. The performance of SWT is confirmed by extensive testing, establishing it as a favored option for ECG signal filtration. The document additionally assesses various methodologies including high-pass filtering, notch filtering, Kalman, SGS, MA, EMD, EWT and SWT filtering. This investigation underscores SWT’s benefits in preserving signal integrity for further examination.

SWT is acknowledged for its capacity to maintain essential cardiac characteristics while efficiently denoising ECG signals. This is essential for precise diagnosis and evaluation of cardiovascular disorders^1,2^. The SWT approach is notably proficient in addressing diverse noise types, including Gaussian and muscular artifact noise, prevalent in ECG data capture^3^. The performance of SWT is improved by its multi-scale analytic capacity, enabling precise signal decomposition and reconstruction while preserving the integrity of the ECG signal^1^.

The capacity of SWT to preserve signal integrity is essential for precise ECG analysis and diagnosis. It efficiently attenuates noise while maintaining the critical characteristics of the ECG signal, rendering it appropriate for clinical applications^2,4^. The method’s resilience to diverse noise sources, such as PLI and muscle artifact noise, renders it a viable option for ECG signal processing^3^ SWT’s efficacy is further corroborated by its enhanced outcomes for signal-to-noise ratio and structural similarity index measure relative to alternative methodologies^4^.

### Comparison with alternative filtration methods

Although SWT is emphasized as an exceptional technique for ECG signal denoising, alternative methods also pro- vide significant benefits. Hybrid methods, such as the amalgamation of wavelet and median filtering, exhibit substantial enhancements in denoising efficacy, indicating that the integration of diverse techniques can augment overall performance. Furthermore, improvements in AI-driven denoising techniques, particularly deep learning models, demonstrate potential for enhancing ECG signal quality and diagnostic precision, especially in wearable and remote monitoring contexts^1^.

The most efficient ECG signal denoising solutions for real-time applications utilize a blend of sophisticated algorithms capable of managing diverse noise types while ensuring computing efficiency. These tactics are essential for guaranteeing precise ECG signal interpretation, which is critical for identifying cardiovascular disorders. The subsequent sections delineate several of the most promising methodologies derived from current research.

High-pass filtering and notch filtering are routinely employed to eliminate baseline wander and PLI, respectively. Nonetheless, their efficacy in maintaining the intricate nuances of the ECG signal may be inferior to that of SWT^5^.

Savitzky-Golay Smoothing filtering is beneficial for data smoothing but may be less effective in addressing intricate noise patterns found in ECG signals^5^.

Moving Average filtering is useful in specific contexts. However, it may not provide the same degree of detail preservation as SWT^6^.

The Adaptive Dual Augmented Extended Kalman Filter (ADAEKF) is a significant advancement in ECG signal denoising. It automatically optimizes hyperparameters and adapts to different noise types, including white Gaussian noise and muscle artifact noise. This method outperforms traditional Kalman filter-based approaches, especially in nonstationary environments with low input SNRs, making it highly effective for real-time applications^7^. A comparative analysis of four methodologies–Discrete Wavelet Transform (DWT), EMD, Kalman Filter, and Kalman Filter Smoother (KFS)-for the removal of 50 Hz PLI from ECG signals was made by Bodile and Talari^8^. The study evaluates each method in terms of output SNR, morphological preservation, and computational cost. Results indicate that the KFS consistently outperforms other techniques, achieving the highest output SNR, closely preserving ECG morphology, and exhibiting low computational overhead. While EMD demonstrates moderate denoising capability, its performance is limited by mode mixing, and DWT shows reduced effectiveness under low SNR conditions. Overall, the Kalman filter-based approaches, particularly KFS, offer a robust and computationally efficient framework for ECG denoising, making them suitable for real-time biomedical applications. Bodile and Rao proposes^9^ a robust, derivative-free framework based on the Cubature Kalman Filter (CKF) for removing noise from ECG signals. Unlike Extended Kalman Filters (EKF), the CKF avoids Jacobian computation and provides a more accurate second-order approximation for non-linear models. The authors implement two variants–CKF3 (with angular velocity in both state and measurement models) and CKF3-2 (angular velocity in state only)–and compare them to their EKF counterparts using signals from the MIT-BIH Normal Sinus Rhythm Database under white Gaussian noise and muscle artifacts across input SNRs from –5dB to 10dB. Results show that CKF consistently outperforms EKF in terms of SNR improvement and robustness to different noise types, although at slightly higher computational cost. The study concludes that CKF-based ECG denoising is a more accurate and stable alternative for processing nonlinear biomedical signals, with future work aimed at incorporating arrhythmic data and exploring square-root CKF variants. A Cubature Kalman Filter (CKF) is a model-based alternative to traditional filtering methods. Unlike the Extended Kalman Filter (EKF), the CKF avoids the need for Jacobian computation and maintains second-order accuracy in non-linear state estimation. The approach utilizes a dynamic model of ECG expressed in a three-state form, incorporating angular velocity either in both the state and measurement models (CKF3) or in the state model alone (CKF3-2). The methodology operates on ECG recordings from the MIT-BIH Normal Sinus Rhythm Database, where synthetic noise such as white Gaussian disturbances and muscle artifacts are applied to assess performance.

A novel quantum smoothing filter, utilizing a penta-diagonal Toeplitz structure, offers a reduction in computational complexity compared to classical filters like discrete wavelet transform. This filter achieves similar accuracy in denoising while significantly reducing gate costs, making it a viable option for real-time ECG signal processing^10^.

Wavelet thresholding methods^11^, particularly those that do not require extensive parameter tuning, have shown effectiveness in real-time denoising. These methods excel in removing real noise types, such as baseline wander and electrode motion noise, which are common in wearable ECG sensors^3^. Additionally, combining wavelet transform with particle swarm optimization (PSO) enhances parameter configuration, improving denoising performance, especially for PLI^2^.

Empirical Mode Decomposition methods are particularly suitable for nonlinear and non-stationary biomedical signals such as ECG, as they adaptively decompose signals without relying on predefined basis functions^12^. Hybrid approaches have also been developed, such as combining Improved Complete Ensemble Empirical Mode Decomposition with Adaptive Noise (ICEEMDAN) and the Quasi-Oppositional Jaya Algorithm (QOJA), which decompose noisy signals into Intrinsic Mode Functions (IMFs), identify those dominated by baseline wander and PLI, and optimize their denoising while preserving clinically significant features^13,14^.

Hybrid methods, such as those combining S-transform, bi-dimensional empirical mode decomposition, and non-local means, provide superior noise suppression and signal preservation. These methods are particularly effective in maintaining the structural integrity of ECG signals^4^. The integration of wavelet transforms with techniques like median filtering has demonstrated superior outcomes in denoising ECG data, suggesting the promise of hybrid methodologies to augment the efficacy of SWT^1^.

Machine learning models, like the Transformer-based Convolutional Denoising AutoEncoder (TCDAE), offer robust denoising capabilities for random mixed noise, with minimal distortion in both time and frequency domains. This model is suitable for clinical applications due to its efficient memory consumption and inference speed^15^. The RunDAE model, a variant of the denoising autoencoder, processes short ECG segments without relying on R-peak detection. It leverages the correlation between consecutive segments, outperforming classical DAE models, particularly in handling motion artifacts and baseline wander^16^.

While these advanced methods provide significant improvements in ECG signal denoising, it is essential to consider the trade-offs between computational complexity and real-time applicability. For instance, while quantum smoothing filters offer reduced complexity, their implementation in real-time systems may still face challenges due to the current state of quantum computing technology. Similarly, machine learning models like TCDAE require substantial training data and computational resources, which may limit their deployment in resource-constrained environments. Therefore, selecting the appropriate denoising strategy should consider the specific application requirements and available computational resources.

## The electrocardiographic signal

The electrocardiographic signal is a critical tool in the non-invasive monitoring and diagnosis of heart conditions. It captures the electrical activity of the heart over time, providing insights into heart rhythms and potential pathologies. The ECG signal is characterized by its waveform, which includes the PQRSTU waveform, and is used to assess various cardiac parameters such as heart rate, rhythm, and the presence of arrhythmias. Recent advancements in ECG signal processing and analysis have enhanced its diagnostic capabilities, particularly through the integration of machine learning and advanced signal processing techniques.

**Fig. 1:**
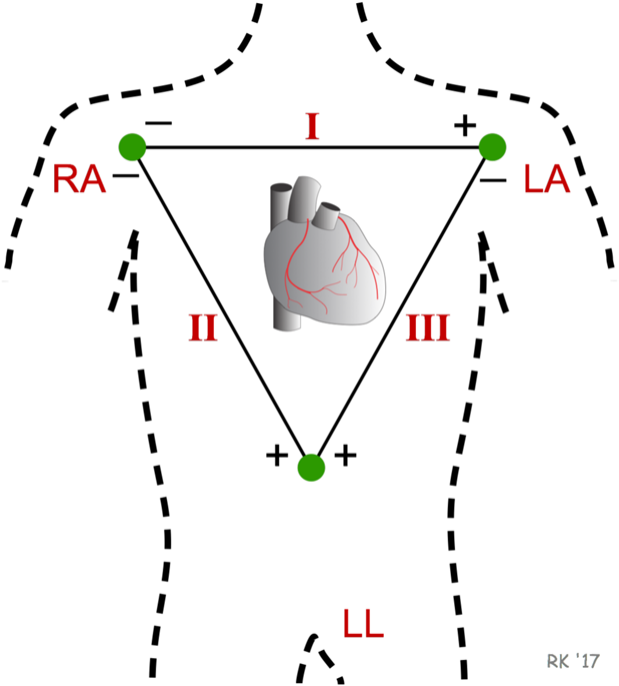
Einthoven’s triangle (Source: https://www.cvphysiology.com/Arrhythmias/A013a).

In Fig. 1, there are several notations, including LA, RA, and LL. These abbreviations stand for left arm, right arm, and left leg. A single ECG lead is the electrical potential difference between two electrodes (Eq. 1). Therefore, the bipolar leads (I, II, III) are given by the following formulas^17^:

1 $$\begin{aligned} \begin{aligned} \text {Lead I}&= \textrm{LA} - \textrm{RA}, \\ \text {Lead II}&= \textrm{LL} - \textrm{RA}, \\ \text {Lead III}&= \textrm{LL} - \textrm{LA}. \end{aligned} \end{aligned}$$

Using leads I, II and III, we can calculate so called augmented leads (Eq. 2 - 4). These are denoted as aVR, aVL, and aVF. The “a” stands for “augmented” and “V” stands for “vector”. Since these leads were constructed using computation, they do not provide any type of new information but rather provide a new view on existing leads. Augmented leads are calculated using the following formulas^18^:

2 $$\begin{aligned} \textrm{aVL}&= \frac{\text {Lead~I} - \text {Lead~III}}{2} \end{aligned}$$

3 $$\begin{aligned} \textrm{aVF}&= \frac{\text {Lead~II} + \text {Lead~III}}{2} \end{aligned}$$

4 $$\begin{aligned} \textrm{aVR}&= -\frac{\text {Lead~I} + \text {Lead~II}}{2} \end{aligned}$$

The electrocardiogram is a weak, non-stationary signal that is affected by a variety of disturbances. These disturbances can significantly impact the quality of the signal, making accurate interpretation challenging. Most of them include^19^:

- Power-line interference - 50/60 Hz harmonics caused by improper patient grounding, electromagnetic interference
- Electrode contact noise - connection between the electrode and patient
- Motion artifact - generated by movement of patient
- Muscle contractions - millivolt noises can be created by voluntary or involuntary muscles
- Baseline wander - noise created by human factors such as breathing, etc. This kind of interference has frequency greater than 1 Hz

The ECG signal spans 0.05–100 Hz, with most energy concentrated below 35 Hz, making it vulnerable to both high-frequency noise (e.g., PLI, EMG) that masks QRS complexes, and low-frequency baseline drift from respiration that overlaps with clinically relevant components. Effective noise suppression is thus essential for reliable diagnosis^20^, yet hardware filtering often introduces distortion due to limited parameter adjustment. To address this, numerous denoising techniques have been developed, including digital filters, wavelet transforms, and empirical mode decomposition.

Denoising ECG signal is a process to separate the valid signal component from undesired signals to obtain noise free ECG that facilitates easy and accurate diagnosis. A denoising approach should detect the different noises in the data and filter the data while ensuring the results obtained are not influenced by undetected artifacts. Some of the approaches used for denoising the ECG signal are adaptive filtering, FIR filtering, fuzzy logic, EMD and EWT^19^.

Electrocardiographic signals tend to be corrupted or distorted during the recording process, especially in cases when ECG is wirelessly recorded; the signal gets corrupted by Additive White Gaussian Noise (AWGN). Over the years, several solutions were introduced for ECG denoising. According to the study^21^, the empirical mode decomposition and nonlocal means (NLM) algorithms are proved to be effective in this case. Moreover, the NLM-based approach is exceeding in retaining the morphological characteristics in comparison to the EMD. In the given study, an effective combination of NLM and EMD was introduced for ECG denoising. However, to reduce the cost of computation, an adjusted version of EMD (M-EMD) was created.

Several studies have been dealing with processing methods to retain the morphology of an ECG signal.

In addition, Kalman filtering has been explored as a dynamic approach to ECG noise reduction. Clifford et al.^22^ demonstrated its efficiency in adaptively suppressing noise in real-time scenarios, especially in cases where the noise characteristics are variable. The Kalman filter not only retains the morphological features of the ECG but also proves highly suitable for applications in wearable and wireless ECG monitoring systems.

The Non-Local Means (NLM) algorithm, introduced by Buades et al.^23^, has been successfully applied to ECG denoising due to its ability to preserve signal morphology while reducing additive white Gaussian noise. Unlike traditional filtering methods, NLM relies on the concept of self-similarity, identifying and averaging similar patterns across the signal to suppress noise without distorting critical features like the P, QRS, and T waves. Experiments have demonstrated that NLM outperforms traditional methods in retaining the morphological characteristics of ECG signals, making it a strong candidate for noise suppression in wireless ECG recordings.

Patro and Kumar^24^ have proposed based FIR filters to adopt for removing low-frequency, mid-frequency and high- frequency noises from the recorded ECG. Various ECG signals from MIT-BIH NSR and ECG ID databases were taken and the results were evaluated using MATLAB. De-noising of the raw ECG signal was done by the configuration of a cascaded FIR filter, which is based on Kaiser Window. This approach reduces all major noises in frequencies ranging from 0 to 100 Hz. Typically, an ECG signal has a bandwidth of 0,1 - 300 Hz range with a typical amplitude of 0.1 to 4 mV. The ECG signal is frequency sensitive and is affected by noise. Considering these facts, low-frequency noises like baseline Wander noise, which has a frequency spectrum from 0,1 to 0,5 Hz, are suppressed in the preliminary stage in preprocessing of the ECG signal. This kind of ECG noise makes both manual and automatic analysis of ECG recording very difficult, especially the ST segment of ECG. These low-frequency elements can also alter the visual interpretation of ECG. The researchers also suggested passing the ECG recording through second cascaded FIR Band stop filter with cutoff frequencies of 59,5 Hz and 60,5 Hz in order to eliminate 60 HZ PLI.

## The risk of improper filtration

In this section, we will discuss and demonstrate the dangers of improper filtration applied to ECG signal. We will conduct a filtration experiment and demonstrate the effects of too much and too little filtration.

The experiment begins by acquiring an ECG signal recorded by SEN0213 chip using Arduino. The data have been recorded on a 26-year-old male patient and saved in csv format. Let’s go ahead and filter it.

**Fig. 2:**
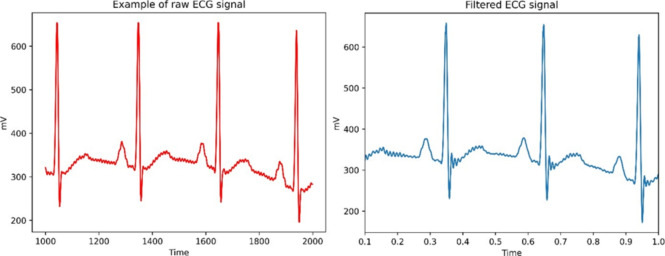
Filtered 50 Hz harmonics from Lead II.

Firstly, we will use Band-stop filter, which combines low-pass and high-pass frequency filtering^25^. We are going set that filter to remove frequencies ranging from 48 – 52 Hz. This approach showed only minor improvement (Fig. 2) in ECG signal. The P wave is smoother, and the isoelectric line is straighter.

**Fig. 3:**
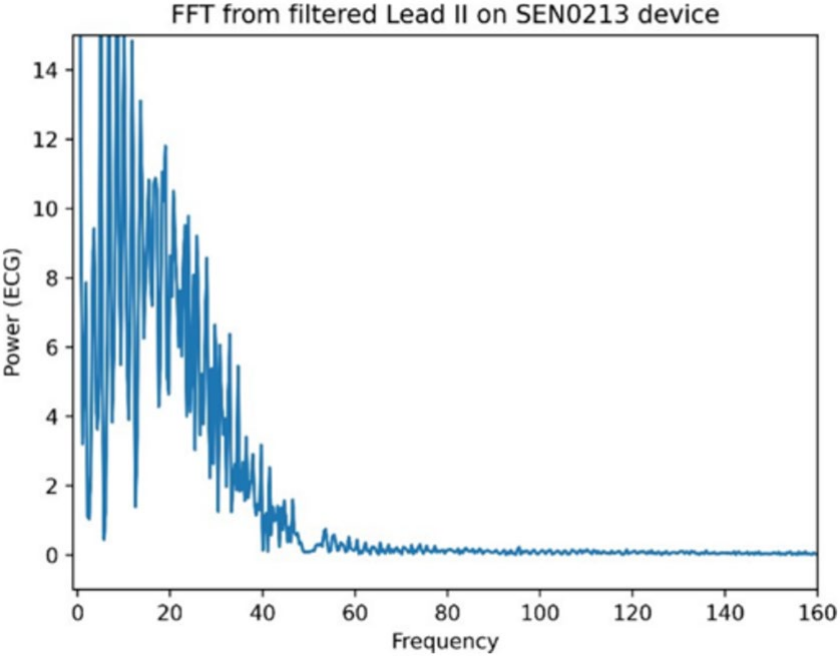
Fourier transformation showing denoised 50 Hz frequency.

We applied Fourier transformation on the filtered signal and have proved that the 50 Hz harmonic signal was successfully removed. Fourier transformation (Fig. 3) was constructed using scipy.fftpack python library.

**Fig. 4:**
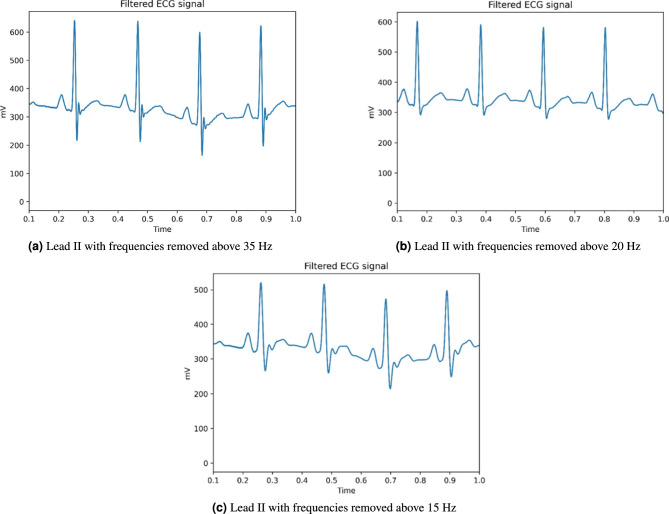
Comparison of Lead II signals with different frequency cutoffs.

If we filter out frequencies above 35 Hz, we can significantly reduce the noise in the ECG signal, leading to a cleaner and more accurate representation of the heart’s electrical activity. By removing these higher frequencies, we eliminate unnecessary artifacts and distortions that can obscure the key components of the ECG, such as the P, QRS, and T waves. The result is presented below (Fig. 4a).

Based on our previous observations, we now suggest eliminating higher frequencies. We are going to conduct several experiments showing different high-pass filtering. The following picture (Fig. 4b) shows Lead II with removed frequencies above 20 Hz. As we can see, the wave morphology remains intact. The ECG signal still retains it’s important parts like P, QRS and T wave.

Finally, we conducted the last experiment with a boundary set to 15 Hz. The resulting signal is becoming severely distorted and unreadable. It is losing its typical ECG characteristics therefore it is not a valid ECG signal. The signal is becoming flattened, the QRS complexes are widening and the T wave is becoming diminished. The result is presented below (Fig. 4c).

**Fig. 5:**
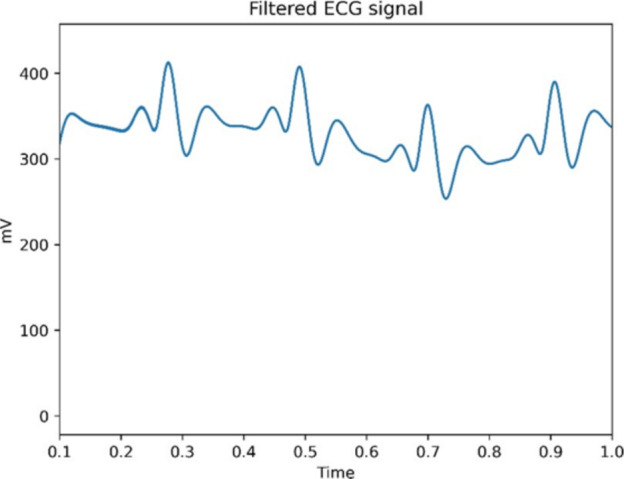
Deformed ECG after excessive filtering.

We can point out that the upper boundary for ECG signal frequency around 20 Hz or 25 Hz covers all the important characteristics of ECG and nothing important drops out. The threshold should not go down below 20 Hz as it deforms the ECG beyond the point of recognition. Below you can see a specific example (Fig. 5) of deformed ECG although you can still imagine and identify the P, QRS and T segments.

During our research, we have concluded that frequencies above 20 Hz are unnecessary for the accurate interpretation of the ECG signal. These higher frequencies often introduce unwanted noise or distortions without contributing meaningful information about the heart’s electrical activity. We have demonstrated that when dealing with ECG filtering, we must be very cautious as to retain the structure and morphology of given ECG signal.

## Filtration experiments using different methods

As mentioned before, the integrity of raw ECG data can be compromised by various forms of noise and artifacts, making accurate data acquisition and effective signal filtering critical phase of any research. The success rate of preprocessing ECGs will be noted via specific metrics such as signal-to-noise ratio and others that are described in the following chapter.

## Introduction to filtration metrics

We have evaluated a comprehensive set of parameters to assess the effectiveness in ECG signal filtering. These metrics played an important role in quantifying noise reduction and preserving the integrity of critical ECG features. The analysis of given parameters is summarized below:

- Mean Squared Error (MSE) and Root Mean Squared Error (RMSE): These metrics highlighted the precision of filtering techniques in approximating the original signal. Lower MSE and RMSE values reflected minimal distortion and excellent signal preservation^26,27^. MSE is a metric used to measure the average squared difference between the estimated values (filtered signal) and the original values (true signal). It is calculated by averaging the squared differences between each point in the filtered signal and the corresponding point in the original signal (Eq. 5).
  5 $$\begin{aligned} \begin{aligned} \text {MSE}(x, \hat{x}) = \frac{\sum _{i=0}^{N - 1} (x[i] - \hat{x}[i])^2}{N} \end{aligned} \end{aligned}$$
  where *N* is the total number of samples. The MSE is particularly useful in ECG signal processing because it quantifies the extent to which a filtering technique alters the original signal. A lower MSE indicates that the filtered signal closely resembles the original signal, which is crucial in maintaining the integrity of ECG features. RMSE is a commonly used metric that measures the square root of the average squared differences between the original signal and the filtered signal. Essentially, it is the square root of the MSE, providing a more interpretable value that is in the same unit as the signal itself. In the context of ECG filtering, RMSE directly reflects how much the filtered signal deviates from the original signal in terms of amplitude (Eq. 6). Lower RMSE values indicate that the filtered signal closely matches the original, which is crucial for preserving the diagnostic features of an ECG.
  6 $$\begin{aligned} \begin{aligned} \text {RMSE}(x, \hat{x}) = \sqrt{\frac{\sum _{i=0}^{N - 1} (x[i] - \hat{x}[i])^2}{N}} \end{aligned} \end{aligned}$$
  where *N* is the total number of samples.
- Signal-to-Noise Ratio (SNR): This parameter demonstrated its utility as a key measure of noise reduction. Higher SNR values consistently correlated with clearer ECG signals, indicating effective noise suppression by advanced filtering methods^28^. It is typically expressed in decibels (dB) and is calculated as the ratio of the power of the true signal($P_{\text {signal}}$) to the power of the noise $P_{\text {noise}}$) present in the data (Eq. 7).
  7 $$\begin{aligned} \begin{aligned} \text {SNR} = 10 \log _{10}\left( \frac{P_{\text {signal}}}{P_{\text {noise}}}\right) \end{aligned} \end{aligned}$$
  where $P_{\text {signal}}$ is the power of the useful signal and $P_{\text {noise}}$ is the power of the noise.
  In denoising problems we have *x* the original signal and $\hat{x}$ the filtered (denoised) signal. Since the true noise is not directly available after filtering, it is estimated as the residual $e = x - \hat{x}.$. The signal energy is approximated by
  $$\begin{aligned} E_{\text {signal}} = \sum _{i} x[i]^2, \end{aligned}$$
  while the residual error (noise + distortion) energy is
  $$\begin{aligned} E_{\text {error}} = \sum _{i} (x[i] - \hat{x}[i])^2. \end{aligned}$$
  By substituting into the SNR definition we obtain
  8 $$\begin{aligned} \begin{aligned} { \textrm{SNR}_{\textrm{filtered}} = 10 \cdot \log _{10} \left( \frac{E_{\text {signal}}}{E_{\text {error}}} \right) }. \end{aligned} \end{aligned}$$
  Interpretation

- If $\hat{x}[n] \approx x[n]$, the error energy is small, therefore $\textrm{SNR}_{\textrm{filtered}}$ is high $\;\Rightarrow$ good filtering.
- If the filter distorts the signal, the error energy grows and $\textrm{SNR}_{\textrm{filtered}}$ decreases.

In the context of ECG signal processing, SNR is mostly relevant because a high SNR indicates that the important features of the ECG signal, such as the P-wave, QRS complex, and T-wave, are preserved while minimizing the effect of noise and artifacts. This metric (Eq. 8) is essential for evaluating the effectiveness of a filtering technique. An increase in SNR after filtering suggests that the method successfully enhanced the signal quality by reducing noise without distorting the critical ECG features. This metric is one of the most commonly used metrics to compare the performance of different filtration techniques.

- Peak Signal-to-Noise Ratio (PSNR): As a relative measure of signal clarity, PSNR proved particularly useful in comparing the performance of different denoising approaches^29^. Peak Signal-to-Noise Ratio (PSNR) is a widely used metric in signal processing that measures the quality of a filtered signal compared to its original version, focusing on the peak intensity. It is expressed in decibels and is particularly useful when the signal has high variability, such as ECG signals. The PSNR compares the maximum possible signal value to the noise introduced by the filtering process, thereby indicating how much noise affects the highest amplitude components of the signal. In the context of ECG filtering, a higher PSNR suggests that the filtering technique effectively preserves the sharp peaks and essential features of the signal while minimizing noise. The PSNR is especially relevant in ECG filtering as it balances the need for noise reduction while ensuring that the peak signal components remain undistorted.
  9 $$\begin{aligned} \begin{aligned} \text {PSNR}(x, \hat{x}) = 10 \cdot \log _{10} \left( \frac{\text {MAX}(x)^2}{\text {MSE}(x, \hat{x})}\right) \end{aligned} \end{aligned}$$
  where *MAX* represents the maximum possible value of the signal, and MSE is the Mean Squared Error between the original and filtered signals
- Percentage Root-Mean-Square Difference (PRD): PRD emphasized the importance of maintaining morphological fidelity in ECG signals. Techniques with lower PRD scores demonstrated their superiority in retaining key cardiac features, essential for accurate diagnosis^30^. Percentage Root-Mean-Square Difference (PRD) is a metric used to assess the distortion level between an original signal and its filtered version. It measures the percentage of error introduced by the filtration process, comparing the filtered signal to the unfiltered ECG (Eq. 10). A low PRD value, typically below 10%, indicates minimal distortion and suggests that the filtration preserves the original signal well. The PRD is calculated using this formula:
  10 $$\begin{aligned} \begin{aligned} \text {PRD} (\%) = \frac{\sqrt{\sum _{i=1}^{N} (x[i] - \hat{x}[i])^2)}}{\sqrt{\sum _{i=1}^{N} (x[i])^2}} \times 100 \end{aligned} \end{aligned}$$
  where *N* is the total number of samples.
- Correlation Coefficient (Pearson’s r): This metric evaluated the relationship between filtered and original signals, with values closer to 1 indicating stronger agreement^31^. The Correlation Coefficient (Pearson’s r) is a statistical measure that quantifies the linear relationship between two variables, ranging from -1 to +1 (Eq. 11). In ECG filtration, it is used to evaluate the similarity between the original ECG signal and the filtered version. A value of +1 indicates a perfect positive correlation, meaning the filtered signal matches the original exactly, while a value of -1 indicates a perfect negative correlation, and a value close to 0 suggests no correlation. The higher the correlation coefficient (close to +1), the better the filtration technique preserves the original ECG features. The formula for calculation is
  11 $$\begin{aligned} \begin{aligned} \text {r}(x, \hat{x}) = \frac{ \sum _{i=0}^{N - 1} ((x[i] - mean(x))*(\hat{x}[i] - mean(\hat{x}))) }{ \sqrt{ \sum _{i=0}^{N - 1} (x[i] - mean(x))^2} *\sqrt{ \sum _{i=0}^{N - 1} (\hat{x}[i] - mean(\hat{x}))^2} } \end{aligned} \end{aligned}$$
  where *N* is the total number of samples.
- Kurtosis and Skewness: These statistical measures provided insights into the shape and symmetry of signal distributions. Balanced kurtosis and reduced skewness indicated better noise handling without introducing artifacts, critical for downstream machine learning applications^32^. In ECG filtration, kurtosis is used to assess the presence of outliers and the peakedness of the signal distribution after filtration. It helps in identifying whether the filtered signal retains the sharp peaks like QRS complex. High kurtosis values indicate more pronounced peaks, which is often desired in ECG signal processing as it suggests better preservation of important features. In the context of ECG filtration, skewness is used to evaluate whether the filtered signal remains symmetric or becomes skewed due to noise or artifacts. The ECG signals typically have certain asymmetries, and preserving the natural skewness is important for accurate clinical analysis. Positive skewness indicates a distribution with a longer tail on the right, while negative skewness indicates a longer tail on the left. A skewness value near zero indicates a symmetric distribution, while positive or negative values indicate right or left skewness. In ECG signal processing, monitoring skewness helps in ensuring that the filtration technique does not excessively alter the signal’s inherent characteristics.
- Standard Deviation (Std Dev): It is a measure of the amount of variation or dispersion in a set of values. In ECG filtration, it is used to assess how much the signal values deviate from the mean. A low standard deviation indicates that the signal values are closely clustered around the mean, while a high standard deviation suggests more variation, which could be due to noise or artifacts. Calculating standard deviation before normalization ensures that we capture the true variability of the raw signal, which is critical for assessing its original quality. After normalization, the standard deviation is artificially standardized, masking any inherent differences and making it less informative for understanding the signal’s characteristics. Thus, in our experiment we will only calculate Std Dev before normalization. This will provide an accurate reflection of the signal’s original state. The Std Dev was used in case of computing SNR directly from the original signal itself by using the formula
  12 $$\begin{aligned} \begin{aligned} {SNR}_{\text {original}} = 20 \cdot \log _{10} \left( \frac{\textrm{Range}(x)}{\sigma _x} \right) , \end{aligned} \end{aligned}$$
  where $\textrm{Range}(x) = \max (x) - \min (x) = \text {peak-to-peak amplitude}, \sigma _x = \textrm{std}(x).$ The signal amplitude is approximated by its peak-to-peak value and the noise level is approximated by the standard deviation of the signal samples (Eq. 12). Thus, the ratio $\frac{\textrm{Range}(x)}{\sigma _x}$ is used as an estimate of the signal-to-noise ratio. The factor 20 (instead of 10) is used because this ratio compares amplitudes rather than powers. This is a heuristic method. It provides a quick estimate of SNR when no clean reference signal is available. It assumes that most of the variance of *x* arises from noise, while the peak-to-peak range corresponds to the underlying useful signal.

## Prerequisites and database used for experiment

In order to effectively conduct the experiments, Python 3.12 was chosen as the programming language due to its strong capabilities and ability to work smoothly with a range of scientific libraries. The most important libraries used for data analysis and machine learning tasks were numpy 2.0.1 for numerical computations, pandas 2.2.2 for data manipulation and analysis, and matplotlib 3.9.1 for data visualization. In addition, the scikit-learn library version 1.5.1 was used to construct and analyze machine learning models, while the scipy library version 1.14.0 was used for scientific and technical computations.

In regard to the ECG databases, we have carefully analyzed several most well-known databases and tried to pick one that would best suit our needs. These following databases were considered:

- PTB Diagnostic ECG Database
- MIT-BIH Arrhythmia Database
- CSE (Common Standards for Electrocardiography) Database
- Chapman-Shaoxing 12-Lead ECG Database
- St. Petersburg INCART 12-lead Arrhythmia Database
- LUDB (Lobachevsky University Database)

After careful consideration, we chose PTB Diagnostic ECG Database due to several compelling reasons:

- **Comprehensive ECG data**: This database offers a solid dataset for diagnostic analysis and machine learning model training since it includes extensive and accurate ECG recordings from both healthy and sick individuals.
- **Standardized format**: Because of the database’s uniform structure, preprocessing data is made easier, and experiment consistency is achieved. For scientific research to be accurate and reproducible, it is crucial to maintain consistency in the data structure. While no formal standards state the format of the records in this particular database, its internal uniform structure ensures consistency and reliability across experiments.
- **Wide acceptance and validation**: The PTB Diagnostic ECG Database is widely accepted and validated within the research community. The fact that it is used in numerous studies and publications highlights its reliability and significance for ECG analysis.
- **Rich metadata**: In addition to the ECG recordings, the database has extensive metadata, including patient demographics, medical history, and diagnosis. This additional information enhances the context and possible discoveries that can be obtained from the data.
- **Support for machine learning**: The range and high quality of the data makes it well-suited for the development and evaluation of machine learning models designed for ECG interpretation and diagnostic purposes.
- **Accessibility**: The database is freely accessible to researchers, providing an essential resource without any obstacles.

The combination of these elements makes the PTB Diagnostic ECG Database a valuable tool for conducting accurate and effective ECG diagnostic studies. In comparison, other databases have limitations that made them less suitable for our study. The MIT-BIH Arrhythmia Database, while highly valuable, primarily focuses on arrhythmias and only includes a limited number of 12-lead recordings, reducing its usability for deeper analysis. The CSE Database seems comprehensive, but requires a paid license, which introduces access barriers, thus limiting its availability for extensive experimentation. The St. Petersburg INCART Database also demands a purchase, and its focus on arrhythmia cases lacks the diversity found in PTB. Finally, while the LUDB and Chapman-Shaoxing databases are freely available, they either impose usage restrictions or lack the extensive clinical variety needed for a robust filtering performance comparison. These factors make the PTB Diagnostic ECG Database (Table 1) with $516 \times 12$ records the most balanced choice for our experimentation.

The ECG dataset appears to be moderately noisy (mean SNR $\sim$16.5 dB), generally flat or smooth (low kurtosis, skewness, IQR, and standard deviation). It contains some sharp peaks or anomalies (as seen in max kurtosis and skewness). It is suitable for further analysis.

**Table 1:** Characteristics of ECG signals from the PTB Diagnostic ECG Database.

Metrices	original				original + 10dB AWGN				original + 15dB AWGN			
	min	max	mean	median	min	max	mean	median	min	max	mean	median
**SNR (dB)**	9.113	35.762	16.490	16.707	13.469	35.381	17.747	17.768	11.751	35.644	17.185	17.279
**Kurtosis**	-1.703	326.748	2.493	0.338	-1.408	270.621	2.060	0.278	-1.598	306.413	2.342	0.317
**Skewness**	-4.864	5.912	0.086	0.045	-4.220	5.131	0.074	0.038	-4.639	5.647	0.082	0.042
**IQR**	0	$4.658{\times }10^{-03}$	$3.889{\times }10^{-04}$	$2.293{\times }10^{-04}$	$2.300{\times }10^{-05}$	$4.330{\times }10^{-03}$	$3.974{\times }10^{-04}$	$2.395{\times }10^{-04}$	$2.000{\times }10^{-05}$	$4.521{\times }10^{-03}$	$3.910{\times }10^{-04}$	$2.325{\times }10^{-04}$
**Std Dev**	$2.874{\times }10^{-05}$	$2.308{\times }10^{-03}$	$2.667{\times }10^{-04}$	$1.795{\times }10^{-04}$	$3.016{\times }10^{-05}$	$2.418{\times }10^{-03}$	$2.798{\times }10^{-04}$	$1.884{\times }10^{-04}$	$2.918{\times }10^{-05}$	$2.343{\times }10^{-03}$	$2.709{\times }10^{-04}$	$1.824{\times }10^{-04}$

To mimic noisy environment we also apply AWGN at a given signal-to-noise ratio ($\text {SNR} \in \left( 10\text {dB}, 15\text {dB} \right)$). In realistic modeling of ECG signals, it is more appropriate to add different AWGN to each channel rather than applying the same noise profile across all channels. This strategy reflects the heterogeneous nature of real-world noise affecting multi-lead ECG recordings. From the physiological point of view each ECG lead represents a unique projection of the heart’s electrical activity. Consequently, external noise sources such as baseline wander, electromyographic (EMG) interference, and PLI artifacts affect each channel differently due to

- Variability in electrode placement and orientation.
- Differences in anatomical and muscular proximity.
- Unequal impedance and lead-specific hardware characteristics.

In case of SNR-aware noise modeling, the signal amplitudes and morphologies vary across channels. For example, limb leads like Lead II often exhibit higher voltages than precordial leads such as V1. To maintain consistent noise perception across channels, it is advisable to compute noise power (Eq. 13) for each channel based on its signal power

13 $$\begin{aligned} \begin{aligned} P_{\text {noise},i} = \frac{P_{\text {signal},i}}{10^{\text {SNR}_{\text {target}} / 10}}, \end{aligned} \end{aligned}$$

where *i* is the channel index and $P_{\text {signal},i}$ is the average power of the signal in channel *i*. In our approach we decide to use different AWGN with the same target SNR, which leaded to independent noise, but with uniform SNR.

### The length of the ECG records

The optimal length of an ECG recording for diagnostic purposes depends on the clinical context and the specific condition being assessed. A standard 12-lead ECG, typically lasting around 10 seconds, is sufficient for evaluating cardiac rhythm, conduction abnormalities, hypertrophy, and signs of ischemia or myocardial infarction. However, in cases of intermittent arrhythmias or syncope, extended monitoring–such as 24- to 48-hour Holter ECG or event recorders lasting days to weeks–is necessary to capture infrequent pathological episodes. For research or advanced signal processing applications, longer recordings ranging from 30 seconds to several minutes are preferable to allow for reliable spectral analysis, wavelet decomposition, or assessment of heart rate variability. The duration of individual recordings in the PTB Diagnostic ECG Database is summarized in Table 2.

**Table 2:** Distribution of record durations in the PTB Diagnostic ECG Database.

**Duration [s]**	**Record Count**
120.012	1356
118.184	12
115.200	4308
115.174	36
97.000	12
96.588	12
91.987	12
87.910	12
76.800	12
38.400	384
32.000	36

In the context of signal filtering, shorter segments of 4 seconds may be adequate for applying basic filters such as high-pass filters, notch filters, SGS, Kalman filtering, or MA filters, since these methods operate effectively on localized time windows. By contrast, moderately longer segments of 8 seconds provide a more stable basis for parametric filters such as Chebyshev type II, where frequency-selective characteristics require sufficient data for accurate filter response.

For more advanced techniques such as SWT, EMD, and EWT, longer segments of at least 8 to 16 seconds are generally recommended to ensure adequate time–frequency resolution and decomposition depth. Specifically, SWT and EWT benefit from segment lengths that are powers of two, enhancing stability and efficiency of multilevel analysis.

Overall, while short ECG segments may suffice for static morphological analysis and simple filtering, longer intervals substantially improve the robustness and accuracy of advanced denoising and decomposition methods.

In our research, we chose to analyze 16.384-second segments (corresponding to $2^{14} = 16{,}384$ samples at 1 kHz sampling rate), extracted individually from each lead of the 12-lead ECG recordings. The start of each segment was fixed at the 10-second mark of the original recording. This decision was made to ensure the exclusion of artifacts, and potential baseline instabilities commonly present at the beginning of ECG recordings, such as patient movement, lead adjustment, or signal clipping during initialization. Starting the analysis after the first 10 seconds provided a more stable and representative segment of the cardiac signal for consistent and comparable filtering and denoising across all datasets (Table 3).

**Table 3:** Characteristics of ECG signals segments from the PTB Diagnostic ECG Database.

Metrices	slice(signal[10:26.384])				slice(signal[10:26.384] + 10dB AWGN)				slice(signal[10:26.384] + 15dB AWGN)			
	min	max	mean	median	min	max	mean	median	min	max	mean	median
**SNR (dB)**	9.141	36.177	17.573	17.783	12.460	35.666	18.641	18.620	10.892	36.003	18.371	18.429
**Kurtosis**	-1.762	386.695	7.112	4.750	-1.369	314.750	4.502	2.227	-1.630	354.389	5.880	3.416
**Skewness**	-6.407	10.979	0.241	0.153	-4.521	8.519	0.155	0.044	-5.026	10.107	0.198	0.093
**IQR**	$1.200\times 10^{-05}$	$2.334\times 10^{-03}$	$1.319\times 10^{-04}$	$8.600\times 10^{-05}$	$1.900\times 10^{-05}$	$2.310\times 10^{-03}$	$1.815\times 10^{-04}$	$1.273\times 10^{-04}$	$1.500\times 10^{-05}$	$2.308\times 10^{-03}$	$1.500\times 10^{-04}$	$1.025\times 10^{-04}$
**Std Dev**	$1.779\times 10^{-05}$	$1.468\times 10^{-03}$	$1.247\times 10^{-04}$	$1.029\times 10^{-04}$	$2.121\times 10^{-05}$	$1.545\times 10^{-03}$	$1.563\times 10^{-04}$	$1.252\times 10^{-04}$	$1.892\times 10^{-05}$	$1.494\times 10^{-03}$	$1.364\times 10^{-04}$	$1.124\times 10^{-04}$

## High-pass filtering

We will start off with high-pass filtering method. We have successfully implemented a python script, that uses high-pass filtering in data from PTB diagnostic database. The code is designed to process application of high-pass filters to remove low-frequency noise like baseline wander. We have automated the resampling, filtering, and analysis of ECG data across multiple patient records while extracting relevant statistical and signal quality metrics for comparison.

When designing a high-pass filter for ECG signal processing, the primary objective is to eliminate baseline wander–typically caused by respiration, patient movement, or electrode drift–without distorting clinically relevant low-frequency components. To achieve this, the filter must attenuate baseline fluctuations in the 0–0.3 Hz range while preserving the morphology of key ECG features: the P wave (0.67–5 Hz), T wave (1–7 Hz), and QRS complex (10–50 Hz). A cutoff frequency of 0.5 Hz is commonly selected, as it strikes a balance between effective baseline drift removal and minimal distortion of diagnostic features.

To identify optimal filter configurations, we experimented with a range of parameters. The cutoff frequency was fixed at 0.5 Hz, which is standard for removing baseline drift in ECG signals. We varied the filter order from 2 to 6 to assess the trade-off between sharpness and signal distortion. Additionally, we tested both normalized and non-normalized filter designs to evaluate their influence on frequency response characteristics. We tried a total of 10 combinations used as input parameters to the filter. This combination of parameters allowed us to systematically evaluate the impact of filter sharpness and normalization on ECG signal quality and morphological preservation. Based on the evaluation results, the optimal configuration was obtained with a cutoff frequency fixed at 0.5 Hz, a filter order of 4 and normalization enabled.

One important aspect when dealing with such filtering is the need for accurate signal resampling, as ECG signals may originally be sampled at various rates, and ensuring a uniform target sampling rate (we used 500 Hz in our code) is crucial for consistent analysis in our experiment. The decision to downgrade the sampling frequency from 1000 Hz to 500 Hz was carefully considered. One of the reasons for this step is that a reduction in the sampling rate decreases the volume of data, which in turn lowers storage requirements and computational demands. Processing a signal sampled at 500 Hz is more efficient, especially in real-time applications, without significantly impacting the quality of the analysis. Additionally, most clinically relevant features in ECG signals, such as the P wave, QRS complex, and T wave, occur within the frequency range of 0.05 to 150 Hz. Since 500 Hz provides a Nyquist frequency of 250 Hz, it is still more than adequate to capture the necessary details of these features without risking frequency misrepresentation (aliasing). Lastly, using a lower sampling rate simplifies signal processing steps, such as filtering and resampling, while maintaining sufficient resolution for accurate clinical interpretation. Thus, the reduction from 1000 Hz to 500 Hz remains a practical choice without compromising the integrity of the ECG analysis. We decide to use Butterworth and zero-phase filtering to protect ECG morphology while effectively removing baseline drift. Butterworth offers a good balance between sharpness and minimal phase distortion. The cutoff frequency was set to 0.5 Hz with filter order 4. After conducting given filtration technique, we have acquired results described below (Table 4).

**Table 4:** Acquired results from high-pass filtering experiment.

Metrices	High-pass(signal)				High-pass(signal + 10dB AWGN)				High-pass(signal + 15dB AWGN)			
	min	max	mean	median	min	max	mean	median	min	max	mean	median
**MSE**	$5.735\times 10^{-11}$	$7.574\times 10^{-06}$	$1.598\times 10^{-07}$	$2.052\times 10^{-08}$	$1.422\times 10^{-10}$	$8.000\times 10^{-06}$	$1.735\times 10^{-07}$	$2.406\times 10^{-08}$	$8.463\times 10^{-11}$	$7.706\times 10^{-06}$	$1.641\times 10^{-07}$	$2.177\times 10^{-08}$
**RMSE**	$7.573\times 10^{-06}$	$2.752\times 10^{-03}$	$2.566\times 10^{-04}$	$1.433\times 10^{-04}$	$1.192\times 10^{-05}$	$2.829\times 10^{-03}$	$2.734\times 10^{-04}$	$1.551\times 10^{-04}$	$9.199\times 10^{-06}$	$2.776\times 10^{-03}$	$2.622\times 10^{-04}$	$1.476\times 10^{-04}$
**PSNR**	-55.565	41.109	7.391	8.405	-55.913	37.943	6.139	7.635	-55.671	39.811	6.875	8.138
**PRD (%)**	5.526	101.224	77.780	89.688	31.714	318.242	85.868	95.583	18.425	193.663	80.580	91.849
**Correlation**	0.041	0.999	0.751	0.854	-0.039	0.961	0.586	0.671	-0.016	0.986	0.663	0.771
**SNR (dB)**	-0.106	25.151	2.936	0.945	-10.055	9.975	1.684	0.392	-5.741	14.692	2.420	0.739
**Kurtosis**	-0.963	678.230	14.276	12.578	-0.748	373.685	5.949	3.755	-0.881	430.228	8.785	6.678
**Skewness**	-23.549	20.209	0.457	0.774	-6.861	9.994	0.194	0.046	-13.008	14.526	0.281	0.131
**IQR**	$6.216\times 10^{-06}$	$8.713\times 10^{-04}$	$4.323\times 10^{-05}$	$3.318\times 10^{-05}$	$1.780\times 10^{-05}$	$1.136\times 10^{-03}$	$1.383\times 10^{-04}$	$1.037\times 10^{-04}$	$1.280\times 10^{-05}$	$9.559\times 10^{-04}$	$9.066\times 10^{-05}$	$7.087\times 10^{-05}$
**Std Dev**	$7.710\times 10^{-06}$	$9.464\times 10^{-04}$	$8.674\times 10^{-05}$	$6.996\times 10^{-05}$	$1.392\times 10^{-05}$	$1.007\times 10^{-03}$	$1.324\times 10^{-04}$	$1.099\times 10^{-04}$	$1.023\times 10^{-05}$	$9.634\times 10^{-04}$	$1.064\times 10^{-04}$	$9.041\times 10^{-05}$

The SNR of filtered ECG segments ranges from 9.141 dB to 36.177 dB, indicating variable initial signal quality, with a mean of 17.573 and median of 17.783 dB (Table 3). After filtering, the High-Passed SNR drops to a mean 2.936 dB and median of 0.945 dB, with values going to 25.151 dB, suggesting that while some signals have improved, others experience significant distortion.

The MSE and RMSE values show moderate errors, with a mean RMSE of $2.566\times 10^{-4}$, reflecting some signal alteration after filtering. The PSNR has a median value of 8.405 dB, indicating that while the filtered signals retain some original information, significant noise or distortion is discovered. The PRD is notably high with a median of 89.688%, further confirming that the filter may be introducing deviations from the original signals.

The Correlation Coefficient between the original and filtered signals has a median of 0.854, indicating very good preservation of the signal’s structure. However, the range (0.041 to 0.999) suggests considerable variation in how well the filter preserves different signals. The Kurtosis and Skewness metrics highlight that certain signals have significant outliers or asymmetry, likely due to high-frequency noise or filtering artifacts.

The Pre-Normalization Standard Deviation, with a median of $6.996\times 10^{-5}$, provides insight into the original variability of the signals before normalization and filtering, showing that some signals had low variability, making them more susceptible to distortion. The IQR metric on the other hand remains relatively stable.

**Fig. 6:**
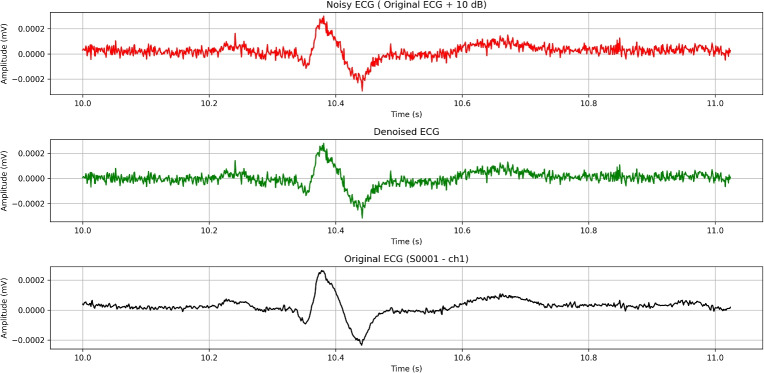
Effect of high-pass filtering on an ECG signal (~1-second ECG segment).

Overall, the metrics suggest that while the high-pass filter is effective at removing low-frequency noise, it introduces significant signal distortion in many cases (Fig. 6).

The high-pass filtering approach demonstrates consistent and robust performance across varying noise levels, with particularly strong resilience under 15 dB AWGN. Compared to 10 dB, 15 dB noise introduces less disruption, as evidenced by improved PSNR, PRD, SNR, and correlation metrics. Overall, the filter effectively preserves ECG waveform morphology and beat-to-beat intervals, with RMSE increasing by less than 7% under noise – a sign of excellent fidelity. Core distortion metrics such as MSE, RMSE, PSNR, PRD, and SNR are tightly interrelated and remain relatively stable, whereas distributional descriptors like kurtosis, skewness, IQR, and standard deviation are highly sensitive to noise, reflecting a normalization effect on signal statistics. Interestingly, the 10 dB noise condition exhibits non-monotonic metric behavior, likely due to its stronger impact on outlier-sensitive measures. While filtering suppresses baseline drift and noise, it may also dampen high-order statistical features essential for anomaly or arrhythmia detection. For diagnostic purposes, metrics should be selected based on clinical relevance–RMSE for QRS detection, correlation for morphological fidelity, and kurtosis for capturing abnormality signatures.

## Chebyshev Type II filtering

Chebyshev Type II filters, also known as inverse Chebyshev filters, are a class of digital and analog filters characterized by an equiripple response in the stopband and a monotonic, ripple-free response in the passband. Unlike Chebyshev Type I filters, which allow ripples in the passband, Type II filters provide a flat passband, making them suitable for applications where signal fidelity within the passband is critical. Their sharp transition between the passband and stopband is achieved at the cost of a potentially higher filter order, which increases computational complexity. The design of these filters typically involves specifying the desired passband edge, stopband edge, and the minimum stopband attenuation, which together determine the order and coefficients of the filter.

For the Chebyshev type II filter, parameter tuning was carried out by evaluating the empirically determined parameter combinations. The cutoff frequency was fixed at 0.5 Hz, the upper cutoff frequency was varied in the range of 25–40 Hz, the filter order was explored from 4 to 8, and the stopband attenuation was adjusted between 40 and 60 dB. The optimal configuration was found to be a lowcut frequency of 0.5 Hz, a highcut frequency of 25 Hz, a filter order of 6, and a stopband attenuation of 40 dB. After applying given filtration technique, we have acquired results described below (Table 5).

**Table 5:** Acquired results from Chebyshev Type II filtering experiment.

Metrices	Chebyshev2(signal)				Chebyshev2(signal + 10dB AWGN)				Chebyshev2(signal + 15dB AWGN)			
	min	max	mean	median	min	max	mean	median	min	max	mean	median
**MSE**	$1.410\times 10^{-10}$	$7.580\times 10^{-06}$	$1.607\times 10^{-07}$	$2.137\times 10^{-08}$	$1.456\times 10^{-10}$	$7.559\times 10^{-06}$	$1.614\times 10^{-07}$	$2.166\times 10^{-08}$	$1.475\times 10^{-10}$	$7.577\times 10^{-06}$	$1.609\times 10^{-07}$	$2.151\times 10^{-08}$
**RMSE**	$1.187\times 10^{-05}$	$2.753\times 10^{-03}$	$2.610\times 10^{-04}$	$1.462\times 10^{-04}$	$1.207\times 10^{-05}$	$2.749\times 10^{-03}$	$2.619\times 10^{-04}$	$1.472\times 10^{-04}$	$1.215\times 10^{-05}$	$2.753\times 10^{-03}$	$2.613\times 10^{-04}$	$1.467\times 10^{-04}$
**PSNR**	-55.573	33.199	6.679	8.225	-55.585	33.149	6.622	8.181	-55.587	33.191	6.662	8.203
**PRD (%)**	10.388	100.446	81.285	91.083	11.637	107.258	81.681	91.385	11.325	101.813	81.404	91.210
**Correlation**	0.014	0.998	0.700	0.788	-0.101	0.996	0.674	0.769	-0.119	0.997	0.689	0.782
**SNR (dB)**	-0.039	19.669	2.224	0.811	-0.609	18.683	2.167	0.782	-0.156	18.919	2.207	0.799
**Kurtosis**	-1.175	65.608	7.850	7.197	-0.939	55.444	6.875	6.055	-1.052	56.252	7.344	6.525
**Skewness**	-6.912	5.135	0.216	0.554	-5.361	5.034	0.163	0.342	-5.783	5.112	0.188	0.460
**IQR**	$5.493\times 10^{-06}$	$9.451\times 10^{-04}$	$4.401\times 10^{-05}$	$3.346\times 10^{-05}$	$8.376\times 10^{-06}$	$1.057\times 10^{-03}$	$5.603\times 10^{-05}$	$4.544\times 10^{-05}$	$6.655\times 10^{-06}$	$9.476\times 10^{-04}$	$4.922\times 10^{-05}$	$3.934\times 10^{-05}$
**Std Dev**	$7.088\times 10^{-06}$	$9.417\times 10^{-04}$	$8.020\times 10^{-05}$	$6.366\times 10^{-05}$	$7.294\times 10^{-06}$	$9.510\times 10^{-04}$	$8.485\times 10^{-05}$	$6.934\times 10^{-05}$	$7.497\times 10^{-06}$	$9.430\times 10^{-04}$	$8.192\times 10^{-05}$	$6.573\times 10^{-05}$

**Fig. 7:**
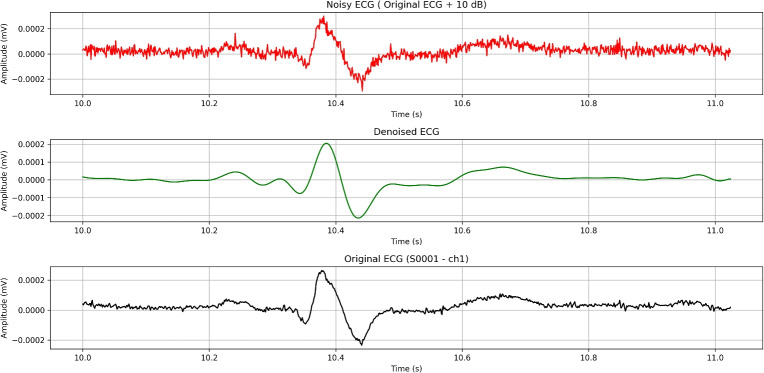
Effect of Chebyshev Type II filtering on an ECG signal (~1-second ECG segment).

The errors remain in a similar range across all noise levels (0 dB, 10 dB, 15 dB). Mean MSE is on the order of $10^{-7}$, while RMSE is around $2.6\times 10^{-4}$. This indicates the filter introduces moderate distortion, independent of added AWGN. The average PSNR is very low (6.6–6.7 dB), with minimum values dipping below –55 dB in some cases. Such low values suggest that the filtered signal differs substantially from the reference signal. Average SNR is very low (2.2 dB). This means residual noise power is still high relative to the signal. The statistical measures (kurtosis, skewness, IQR, standard deviation) suggest that the filter reduces variability but introduces outliers and flattening effects, altering the statistical profile of the ECG signal; the IQR and Std Dev are consistent across noise levels, suggesting the filter strongly compresses the variability of the ECG (Fig. 7).

## Kalman filtering

The Kalman filter is a recursive optimal estimator usable in biomedical signal processing for denoising and feature preservation. In the context of ECG, the measured signal often contains various sources of noise, including PLI, baseline wander, and motion artifacts, which can obscure critical features such as P, QRS, and T waves. The Kalman filter models the ECG as a dynamic system with an underlying “true” signal (state) that evolves over time, while the observed measurements are considered noisy reflections of this state. By recursively combining predictions from the system model with the noisy observations, weighted according to their respective uncertainties, the Kalman filter provides an accurate estimate of the clean ECG signal. Its adaptability allows for effective suppression of both high-frequency and low-frequency noise, while preserving rapid transitions inherent in cardiac events. Proper selection of the filter parameters, including process and measurement noise covariances, is essential to balance noise reduction with signal fidelity, making the Kalman filter a powerful tool for real-time ECG signal enhancement.

For the Kalman filter, parameter tuning was performed by evaluating all combinations of the selected parameters. The constant term was fixed at const = 1, while the process noise covariance (pnc) was varied over the range 1–100 and the measurement noise covariance (mnc) over the range 1–10. This systematic grid search produced a comprehensive set of candidate configurations. The optimal configuration was determined to be (const = 1, pnc = 0.0001, mnc = 0.01), which provided the best filtering performance according to the evaluation criteria.

The performance of the Kalman filter on ECG signals demonstrates its high efficacy in preserving signal fidelity while suppressing noise. Analysis of error metrics (Table 6) shows that the MSE remains extremely low across all noise levels, ranging from $1.892 \times 10^{-9}$ for clean signals to $2.557 \times 10^{-9}$ for 10 dB AWGN, with corresponding RMSE around $3.7 \times 10^{-5}$ to $4.5 \times 10^{-5}$. The PSNR and PRD further support these findings, with median PSNR values around 21 dB and median PRD approximately 20%, indicating that the filtered signals remain close to the original waveforms with minimal distortion.

**Table 6:** Acquired results from Kalman filtering experiment.

Metrices	Kalman(signal)				Kalman(signal + 10dB AWGN)				Kalman(signal + 15dB AWGN)			
	min	max	mean	median	min	max	mean	median	min	max	mean	median
**MSE**	$6.758\times 10^{-12}$	$5.439\times 10^{-08}$	$1.892\times 10^{-09}$	$9.962\times 10^{-10}$	$1.387\times 10^{-11}$	$5.997\times 10^{-08}$	$2.557\times 10^{-09}$	$1.543\times 10^{-09}$	$8.995\times 10^{-12}$	$5.655\times 10^{-08}$	$2.102\times 10^{-09}$	$1.226\times 10^{-09}$
**RMSE**	$2.600\times 10^{-06}$	$2.332\times 10^{-04}$	$3.741\times 10^{-05}$	$3.156\times 10^{-05}$	$3.725\times 10^{-06}$	$2.449\times 10^{-04}$	$4.462\times 10^{-05}$	$3.928\times 10^{-05}$	$2.999\times 10^{-06}$	$2.378\times 10^{-04}$	$4.025\times 10^{-05}$	$3.502\times 10^{-05}$
**PSNR**	-33.216	46.776	20.405	21.790	-34.473	35.311	18.665	20.802	-33.599	38.539	19.578	21.401
**PRD (%)**	0.555	70.043	21.937	19.833	4.839	76.042	23.694	21.437	2.833	70.193	22.574	20.352
**Correlation**	0.606	1.000	0.929	0.934	0.366	0.998	0.910	0.916	0.566	0.999	0.922	0.928
**SNR (dB)**	3.093	45.119	15.950	14.052	2.379	26.306	14.210	13.377	3.074	30.955	15.124	13.828
**Kurtosis**	-1.747	39.409	4.475	2.627	-1.726	38.984	4.279	2.443	-1.744	39.165	4.408	2.549
**Skewness**	-5.076	5.467	0.206	0.148	-5.015	5.390	0.196	0.133	-5.073	5.448	0.203	0.144
**IQR**	$1.076\times 10^{-05}$	$2.334\times 10^{-03}$	$1.314\times 10^{-04}$	$8.603\times 10^{-05}$	$1.248\times 10^{-05}$	$2.332\times 10^{-03}$	$1.344\times 10^{-04}$	$8.935\times 10^{-05}$	$1.160\times 10^{-05}$	$2.324\times 10^{-03}$	$1.323\times 10^{-04}$	$8.726\times 10^{-05}$
**Std Dev**	$1.716\times 10^{-05}$	$1.468\times 10^{-03}$	$1.166\times 10^{-04}$	$9.353\times 10^{-05}$	$1.738\times 10^{-05}$	$1.472\times 10^{-03}$	$1.188\times 10^{-04}$	$9.570\times 10^{-05}$	$1.721\times 10^{-05}$	$1.470\times 10^{-03}$	$1.173\times 10^{-04}$	$9.422\times 10^{-05}$

Correlation analysis reveals high mean values ($\approx 0.91-0.93$) and median values around 0.92-0.93, confirming strong morphological preservation of the ECG signal. The mean SNR is approximately 15 dB, with medians around 13-14 dB, demonstrating effective noise suppression even under varying AWGN conditions.

Statistical measures, including kurtosis (mean $\approx 4.3-4.5$, median $\approx 2.4-2.6$) and skewness (mean $\approx 0.2$, median $\approx 0.13-0.15$), indicate that the signal retains its characteristic peaks and slight asymmetry, while IQR and standard deviation values show that the signal variability and dynamic range are preserved (median IQR $\approx 8.7 \times 10^{-5}$, median Std Dev $\approx 9.35 \times 10^{-5}$).

Finally, the metrics (Table 6) exhibit minimal variation across different noise levels (0 dB, 10 dB, 15 dB), highlighting the Kalman filter’s robustness and adaptive capacity to maintain ECG signal integrity under both clean and noisy conditions. Collectively, these results confirm that the Kalman filter provides reliable denoising while maintaining both the morphological and statistical characteristics of the ECG waveform.

**Fig. 8:**
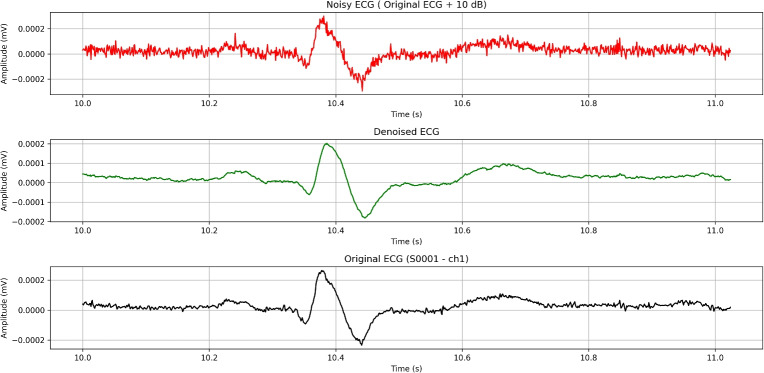
Effect of Kalman filtering on an ECG signal (~1-second ECG segment).

The Kalman filter demonstrates high signal fidelity and effective noise suppression in ECG signals (Fig. 8). Compared to other filters like Chebyshev II, it maintains low MSE/RMSE, moderate PRD, high correlation, and stable SNR, even under added noise. Statistical measures indicate that the essential ECG waveform characteristics, variability, and morphology are preserved, making the Kalman filter a robust choice for ECG denoising in both clean and noisy conditions.

## Moving average filtering

The simple moving-average filter is prized in ECG preprocessing for its remarkable simplicity and efficiency: at each sample it requires just one addition, one subtraction, and one division, making it exceptionally lightweight for real-time or embedded applications. The simple moving-average filter computes each output sample as the mean of the last N input samples. In ECG preprocessing, this sliding-window averaging suppresses high-frequency noise such as muscle artifacts and PLI. In addition to smoothing high-frequency noise, the MA filter can serve as a baseline estimator–using a much longer window–to subtract slow drift without resorting to more complex high-pass designs. Choosing the number of samples span 20–100 samples effectively smooths noise while preserving the sharp QRS complex. This strikes a good balance between noise attenuation and feature preservation. Also, in case of the QRS detection and heartbeat classification, choosing 20 samples (50 ms) is generally optimal.

The application of the moving average filter with a horizon of 20 to the ECG signals demonstrates effective smoothing while largely preserving the intrinsic waveform characteristics.

The error metrics (Table 7) indicate minimal distortion, with mean MSE values ranging from $2.829\times 10^{-9}$ (0 dB) to $3.492\times 10^{-9}$ (10 dB) and median RMSE values below $4.2\times 10^{-5}$, confirming low deviation from the original signal.

**Table 7:** Acquired results from Moving Average filtering experiment.

Metrices	MA(signal)				MA(signal + 10dB AWGN)				MA(signal + 15dB AWGN)			
	min	max	mean	median	min	max	mean	median	min	max	mean	median
**MSE**	$9.547\times 10^{-12}$	$7.501\times 10^{-08}$	$2.829\times 10^{-09}$	$1.495\times 10^{-09}$	$1.647\times 10^{-11}$	$7.558\times 10^{-08}$	$3.492\times 10^{-09}$	$2.128\times 10^{-09}$	$1.191\times 10^{-11}$	$7.589\times 10^{-08}$	$3.039\times 10^{-09}$	$1.736\times 10^{-09}$
**RMSE**	$3.090\times 10^{-06}$	$2.739\times 10^{-04}$	$4.586\times 10^{-05}$	$3.866\times 10^{-05}$	$4.058\times 10^{-06}$	$2.749\times 10^{-04}$	$5.226\times 10^{-05}$	$4.613\times 10^{-05}$	$3.452\times 10^{-06}$	$2.755\times 10^{-04}$	$4.831\times 10^{-05}$	$4.166\times 10^{-05}$
**PSNR**	-35.178	44.917	18.630	19.890	-35.802	34.414	17.277	19.159	-35.445	38.103	18.012	19.644
**PRD (%)**	0.688	83.006	27.029	24.181	4.874	83.381	28.542	25.339	2.911	83.086	27.567	24.584
**Correlation**	0.520	1.000	0.890	0.902	0.357	0.998	0.872	0.885	0.519	0.999	0.884	0.896
**SNR (dB)**	1.618	43.242	14.175	12.331	1.579	26.242	12.823	11.924	1.609	30.718	13.557	12.187
**Kurtosis**	-1.734	41.076	5.167	3.274	-1.713	40.503	4.953	3.056	-1.734	40.757	5.094	3.180
**Skewness**	-5.221	5.48	0.181	0.141	-5.170	5.447	0.172	0.122	-5.216	5.460	0.178	0.134
**IQR**	$1.073\times 10^{-05}$	$2.335\times 10^{-03}$	$1.316\times 10^{-04}$	$8.604\times 10^{-05}$	$1.235\times 10^{-05}$	$2.331\times 10^{-03}$	$1.346\times 10^{-04}$	$8.964\times 10^{-05}$	$1.150\times 10^{-05}$	$2.331\times 10^{-03}$	$1.325\times 10^{-04}$	$8.737\times 10^{-05}$
**Std Dev**	$1.735\times 10^{-05}$	$1.468\times 10^{-03}$	$1.200\times 10^{-04}$	$9.760\times 10^{-05}$	$1.756\times 10^{-05}$	$1.472\times 10^{-03}$	$1.221\times 10^{-04}$	$9.963\times 10^{-05}$	$1.740\times 10^{-05}$	$1.470\times 10^{-03}$	$1.207\times 10^{-04}$	$9.828\times 10^{-05}$

**Fig. 9:**
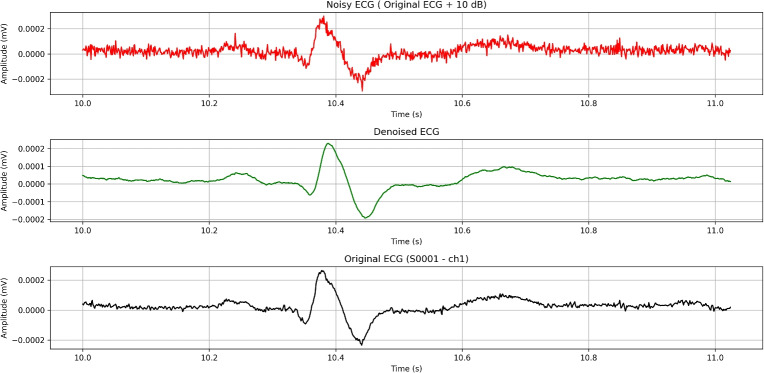
Effect of Moving average filtering on an ECG signal (~1-second ECG segment).

PSNR and PRD further support the fidelity of the filtered signals, with median PSNR around $19-20$ dB and PRD approximately 24-25%, indicating that the overall morphology is well-maintained. Correlation coefficients remain high (median $0.896-0.902$), reflecting strong alignment with the original ECG, while SNR values exhibit slight reductions for noisy segments (median $\approx 12-12.3$ dB), consistent with the smoothing of high-frequency components. Statistical measures reveal a moderate reduction of extreme values and sharp peaks, with median kurtosis decreasing from 4.750 in the original clean segments to $3.18-3.27$, while skewness remains low ($0.13-0.14$), indicating preserved signal symmetry. Additionally, IQR and standard deviation medians decrease slightly, confirming attenuation of local variability. The MA filter performance remains robust under added noise (10 dB and 15 dB AWGN), as evidenced by minimal changes in MSE, PRD, correlation, and SNR. Overall, the MA filter effectively smooths the ECG signals (Fig. 9), reduces high-frequency fluctuations, and preserves essential waveform features, making it suitable for preprocessing in subsequent ECG analysis.

## Notch filtering

Notch filtering, also referred to as band-stop or band-reject filtering, is commonly employed in ECG signal processing to suppress PLI, typically at 50 Hz in Europe and Asia or 60 Hz in North America. In certain cases, harmonics such as 100 Hz or 120 Hz may also require attenuation. The effectiveness of this approach strongly depends on the correct specification of the filter parameters, particularly the target frequency and the quality factor. An inappropriate configuration may lead either to insufficient suppression of the interference or to undesirable distortion of clinically relevant ECG components, such as the QRS complex.

For ECG applications, a 2nd-order biquad notch filter is generally sufficient, and the design must be adapted to the sampling frequency of the dataset (in our case 1000 Hz) to ensure accurate frequency placement. The quality factor *Q* determines the notch bandwidth and is critical for balancing noise removal with signal fidelity. Higher values correspond to narrower bandwidths and therefore more selective filtering. In practice, values of $Q \in \langle 20;35\rangle$ have been shown to achieve effective suppression of PLI without adversely affecting the morphology of the ECG.

In this study, we employed a notch filter with a center frequency fixed at 50 Hz and systematically varied the quality factor (Q) between 25 and 35 to assess the balance between interference suppression and signal fidelity. Based on empirical evaluation, we selected a quality factor of Q = 26, which provides an effective compromise between notch sharpness and robustness to frequency drift. At this setting, the filter targets a narrow band approximately 1.92 Hz wide, centered at 50 Hz. This configuration is well-suited for ECG denoising, as it enables precise attenuation of PLI without introducing significant distortion to nearby signal components. Specifically, the filter attenuates frequencies in the range of approximately 48.04 Hz to 51.96 Hz, ensuring focused suppression of the 50 Hz noise while preserving clinically relevant features such as the P and T waves.

Starting with the SNR (Table 8), the notch-filtered SNR value is markedly high, with a mean of 42.114 dB, indicating improved signal quality, reduced residual noise power, and enhanced clinical usability. The low mean MSE of $6.920E-12$, together with a high median PSNR of 47.796 dB, confirms that the notch filter introduces negligible distortion and effectively preserves the integrity of the original ECG signal. The RMSE remains low, with a mean of $2.003\times 10^{-6}$, suggesting only minor deviations introduced by the filtering process. The PRD metric, with a median of 0.841 %, falls well within acceptable clinical thresholds, showing that the filtered signal closely approximates the original waveform. Furthermore, the correlation coefficient exhibits a mean of 0.999, strongly indicating that the morphological characteristics of the signal are retained. The skewness, with a mean of 0.240, and the kurtosis, with a mean of 7.074, suggest the presence of occasional high-amplitude outliers–typical of physiological signals such as ECG–while still maintaining the overall signal structure. Finally, the IQR value, with a median of $8.605\times 10^{-5}$, alongside a consistent standard deviation, confirms that the variability and dynamic range of the ECG signal are well-maintained across the leads post-filtering.

**Table 8:** Acquired results from notch filtering experiment

Metrices	Notch(signal)				Notch(signal + 10dB AWGN)				Notch(signal + 15dB AWGN)			
	min	max	mean	median	min	max	mean	median	min	max	mean	median
**MSE**	$1.614\times 10^{-14}$	$1.205\times 10^{-09}$	$6.920\times 10^{-12}$	$2.458\times 10^{-12}$	$8.252\times 10^{-11}$	$5.347\times 10^{-07}$	$1.313\times 10^{-08}$	$3.200\times 10^{-09}$	$2.597\times 10^{-11}$	$1.635\times 10^{-07}$	$4.152\times 10^{-09}$	$1.019\times 10^{-09}$
**RMSE**	$1.270\times 10^{-07}$	$3.472\times 10^{-05}$	$2.003\times 10^{-06}$	$1.568\times 10^{-06}$	$9.084\times 10^{-06}$	$7.312\times 10^{-04}$	$8.405\times 10^{-05}$	$5.656\times 10^{-05}$	$5.096\times 10^{-06}$	$4.044\times 10^{-04}$	$4.733\times 10^{-05}$	$3.192\times 10^{-05}$
**PSNR**	-12.418	78.723	46.569	47.796	-44.614	40.863	14.710	16.628	-39.609	45.638	19.679	21.604
**PRD (%)**	0.015	48.863	1.297	0.841	14.275	308.055	31.772	30.073	8.038	174.238	17.939	16.952
**Correlation**	0.860	1.000	0.999	1.000	0.097	0.983	0.825	0.888	0.181	0.994	0.919	0.960
**SNR (dB)**	6.220	76.405	42.114	41.508	-9.773	16.908	10.255	10.436	-4.823	21.897	15.224	15.416
**Kurtosis**	-1.759	385.717	7.074	4.718	-1.370	313.952	4.491	2.223	-1.629	353.721	5.857	3.400
**Skewness**	-6.267	10.777	0.240	0.152	-4.526	8.364	0.154	0.045	-5.027	9.917	0.198	0.094
**IQR**	$1.221\times 10^{-05}$	$2.334\times 10^{-03}$	$1.318\times 10^{-04}$	$8.605\times 10^{-05}$	$1.901\times 10^{-05}$	$2.312\times 10^{-03}$	$1.811\times 10^{-04}$	$1.270\times 10^{-04}$	$1.489\times 10^{-05}$	$2.308\times 10^{-03}$	$1.498\times 10^{-04}$	$1.022\times 10^{-04}$
**Std Dev**	$1.778\times 10^{-05}$	$1.468\times 10^{-03}$	$1.246\times 10^{-04}$	$1.028\times 10^{-04}$	$2.118\times 10^{-05}$	$1.544\times 10^{-03}$	$1.560\times 10^{-04}$	$1.250\times 10^{-04}$	$1.890\times 10^{-05}$	$1.494\times 10^{-03}$	$1.363\times 10^{-04}$	$1.123\times 10^{-04}$

When 10 dB and 15 dB AWGN is introduced, the filter exhibits moderate resilience: although the MSE increases by more than three orders of magnitude at 10 dB (to $1.313 \times 10^{-8}$), the metrics improve considerably at 15 dB (MSE $\approx 4.152 \times 10^{-9}$), PSNR $\approx 19.7$ dB) Nonlinear and statistical descriptors such as kurtosis, skewness, IQR, and standard deviation reflect a flattening and slight symmetrization of the signal distribution with increasing noise, with kurtosis dropping from a clean-signal mean of 7.07 to 4.49 and 5.86 for 10 dB and 15 dB noise, respectively. Correlation remains high across all cases, dropping from 0.999 in clean signals to 0.825 and 0.919 under 10 dB and 15 dB noise, respectively, while percentage root difference (PRD) increases nonlinearly with noise intensity.

**Fig. 10:**
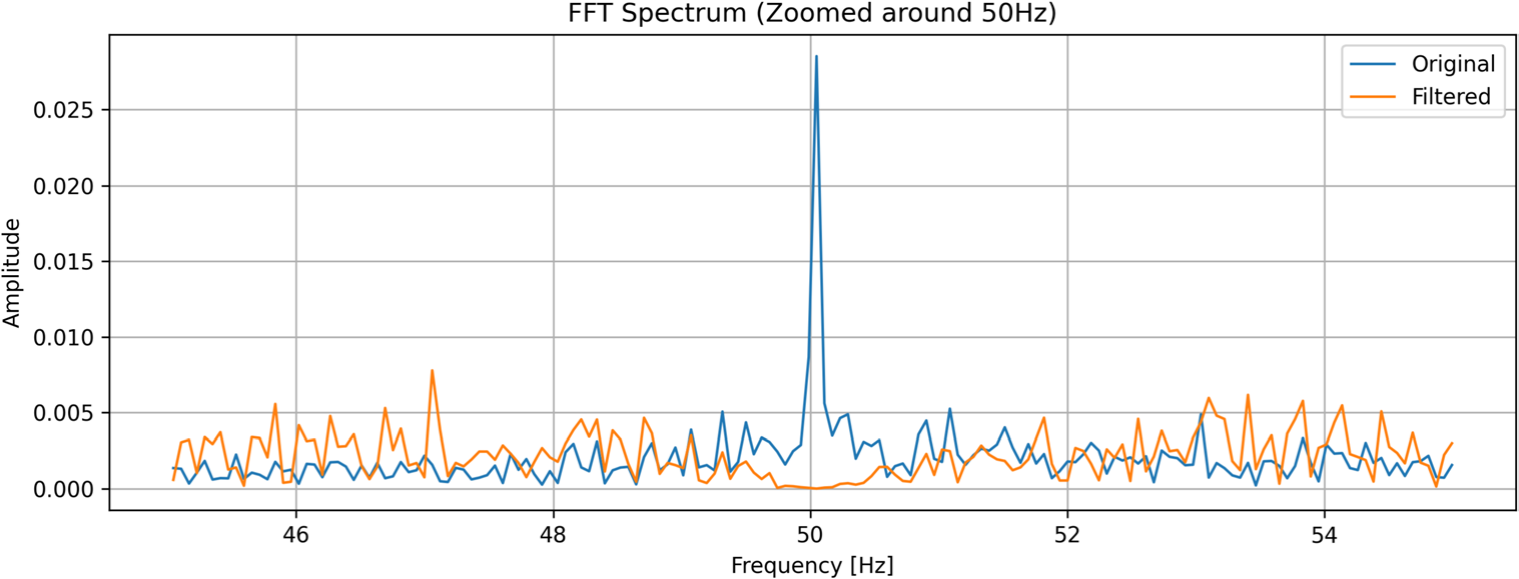
FFT spectrum after using the notch filter on an ECG signal.

Overall, these metrics indicate that the notch filter is effective at selectively removing targeted noise (50 Hz) while preserving the signal’s essential features (Fig. 10).

**Fig. 11:**
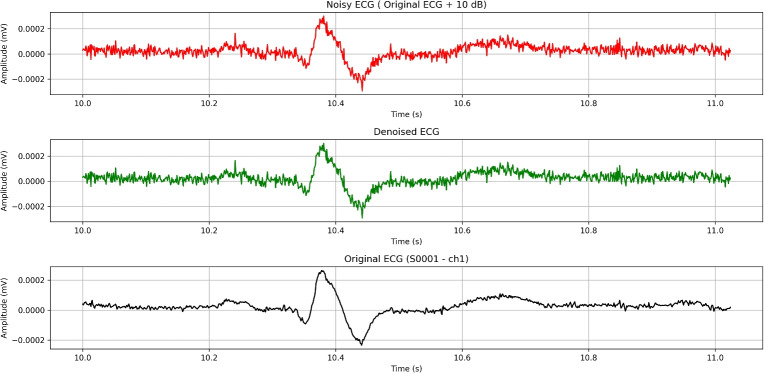
Effect of notch filtering on an ECG signal (~1-second ECG segment).

Despite the high SNR values, alongside the low MSE and high correlation, a visual comparison of the signals shows (Fig. 11) that other noises remain untouched.

## Savitzky-Golay smoothing filtering

The SGS filter is often used for smoothing while maintaining the shape of the given signal. One of the most important considerations when applying the SGS filter was to ensure that the filter’s parameters - particularly the window length and polynomial order - were carefully tuned to balance noise reduction with waveform preservation. For this specific reason, the window length needed to be odd and significantly larger than the polynomial order of 2 to achieve a smooth signal without compromising peak detection accuracy.

To identify the optimal parameters for this filter, the window length was varied over odd values from 9 to 97, the polynomial order was varied from 1 to 8 (ensuring it remained smaller than the window length), and the boundary handling mode was tested across five options: ’mirror’, ’constant’, ’nearest’, ’wrap’, and ’interp’. This search resulted in more than two thousand unique parameter combinations, ensuring that all feasible configurations were evaluated and providing a comprehensive exploration of the parameter space for optimal smoothing performance. The optimal configuration was determined to be a window length of 35, a polynomial order of 3, and a mirror mode, which provided the best smoothing performance according to the evaluation criteria.

The application of the SGS filter to the ECG signal demonstrates high fidelity preservation of the original waveform while effectively reducing high-frequency noise.

Error metrics (Table 9) indicate minimal distortion, with mean MSE values as low as $1.182\times 10^{-10}$ (0 dB) and median RMSE below $7.5\times 10^{-6}$, confirming that deviations from the original signal are negligible.

**Table 9:** Acquired results from Savitzky-Golay Smoothing filtering experiment.

Metrices	SGS(signal)				SGS(signal + 10dB AWGN)				SGS(signal + 15dB AWGN)			
	min	max	mean	median	min	max	mean	median	min	max	mean	median
**MSE**	$1.450\times 10^{-12}$	$1.081\times 10^{-08}$	$1.182\times 10^{-10}$	$5.572\times 10^{-11}$	$1.046\times 10^{-11}$	$3.414\times 10^{-08}$	$9.716\times 10^{-10}$	$3.261\times 10^{-10}$	$4.157\times 10^{-12}$	$1.122\times 10^{-08}$	$3.875\times 10^{-10}$	$1.648\times 10^{-10}$
**RMSE**	$1.204\times 10^{-06}$	$1.040\times 10^{-04}$	$8.865\times 10^{-06}$	$7.465\times 10^{-06}$	$3.234\times 10^{-06}$	$1.848\times 10^{-04}$	$2.431\times 10^{-05}$	$1.806\times 10^{-05}$	$2.039\times 10^{-06}$	$1.059\times 10^{-04}$	$1.612\times 10^{-05}$	$1.284\times 10^{-05}$
**PSNR**	-22.361	57.408	32.729	33.876	-33.106	38.572	24.783	26.979	-28.691	41.153	27.899	30.221
**PRD (%)**	0.166	70.870	5.519	4.413	4.615	79.239	10.296	9.282	2.786	71.027	7.571	6.483
**Correlation**	0.582	1.000	0.994	0.997	0.340	0.998	0.972	0.985	0.566	0.999	0.987	0.992
**SNR (dB)**	2.991	55.594	28.275	27.106	2.021	26.716	20.328	20.647	2.972	31.101	23.445	23.764
**Kurtosis**	-1.723	43.374	6.040	4.018	-1.693	42.462	5.742	3.740	-1.721	42.960	5.937	3.884
**Skewness**	-5.496	5.740	0.203	0.145	-5.437	5.691	0.192	0.124	-5.485	5.711	0.199	0.143
**IQR**	$1.083\times 10^{-05}$	$2.332\times 10^{-03}$	$1.317\times 10^{-04}$	$8.599\times 10^{-05}$	$1.244\times 10^{-05}$	$2.336\times 10^{-03}$	$1.356\times 10^{-04}$	$9.049\times 10^{-05}$	$1.165\times 10^{-05}$	$2.334\times 10^{-03}$	$1.329\times 10^{-04}$	$8.765\times 10^{-05}$
**Std Dev**	$1.753\times 10^{-05}$	$1.468\times 10^{-03}$	$1.232\times 10^{-04}$	$1.014\times 10^{-04}$	$1.778\times 10^{-05}$	$1.473\times 10^{-03}$	$1.258\times 10^{-04}$	$1.036\times 10^{-04}$	$1.759\times 10^{-05}$	$1.470\times 10^{-03}$	$1.240\times 10^{-04}$	$1.022\times 10^{-04}$

**Fig. 12:**
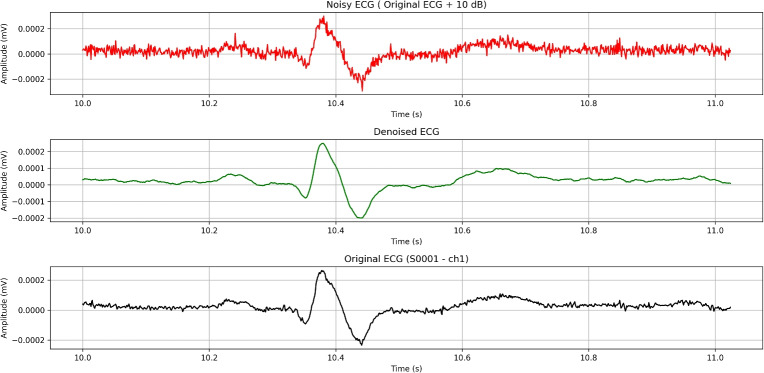
Effect of Savitzky-Golay Smoothing filtering on an ECG signal (~1-second ECG segment).

PSNR values (median 33.876 dB for 0 dB, 26.979 dB for 10 dB) and low PRD percentages (median 4.413–9.282%) further corroborate that the essential ECG morphology is well maintained across varying noise levels. Correlation coefficients remain very high (median 0.997–0.992), indicating near-perfect alignment with the original ECG segments, and SNR values increase substantially (median 27.106 dB for clean signals), reflecting enhanced signal quality relative to background noise. Statistical measures indicate that the SG filter preserves the overall amplitude distribution: median kurtosis decreases modestly from 4.018 to 3.884, suggesting slight attenuation of extreme peaks without altering the overall waveform shape, while skewness remains low (0.124–0.145), confirming the symmetry of the signal is retained. Measures of dispersion, including IQR and standard deviation, exhibit minor reductions, indicating controlled smoothing without significant loss of local variability. Across added noise conditions (10 dB and 15 dB AWGN), the filter maintains robust performance, with only slight changes in MSE, PRD, correlation, and SNR. Overall, the SGS filter provides effective smoothing (Fig. 12), retains critical ECG features, and is particularly suitable for preprocessing in high-precision ECG analyses. However, its performance may vary depending on the noise characteristics and complexity of the signal. In scenarios where precise peak preservation and low distortion are crucial, this method proves to be reliable, but further enhancements may be needed for more aggressive noise reduction without compromising signal accuracy.

## Empirical mode decomposition filtering

Empirical Mode Decomposition (EMD) is a data-driven and adaptive signal processing technique designed to decompose non-linear and non-stationary signals into a finite set of components known as Intrinsic Mode Functions (IMFs). Unlike traditional filtering methods that rely on predefined basis functions, EMD derives its basis adaptively from the signal itself, making it particularly suitable for complex biomedical signals such as ECGs.

In ECG denoising applications, early IMFs typically represent high-frequency artifacts, including muscle noise and PLI, whereas middle IMFs contain the clinically relevant structures such as the P wave, QRS complex, and T wave. Effective denoising requires careful selection of IMFs for signal reconstruction, typically excluding both the highest and lowest frequency components. While EMD does not involve traditional filter parameters as in the previously mentioned filter methods, its performance is influenced by several tunable factors.

For the EMD–based filter, parameter tuning was performed by evaluating the effect of the upper cutoff frequency while keeping the lower cutoff fixed. Specifically, the lowcut frequency was fixed at 0.5 Hz, and the highcut frequency was varied over the range 25–40 Hz. For each combination, the input signal was decomposed into its IMFs using EMD, and a bandpass filter with the specified lowcut and highcut frequencies was applied individually to each IMF. The filtered signal was then reconstructed by summing the filtered IMFs and adding the residual component.

This approach allowed a comprehensive exploration of the upper cutoff parameter, ensuring that the resulting EMD-based filter preserved key signal components while attenuating undesired frequency content. The optimal configuration was determined to be (lowcut = 0.5 Hz, highcut = 36 Hz), which provided the best filtering performance.

The EMD filter demonstrates adaptive denoising performance, with results varying according to noise levels. Error metrics indicate that distortion remains relatively low (Table 10). The MSE is on the order of $10^{-8}$ (mean values), and RMSE medians between $2.0\times 10^{-6}$ (0 dB) and $3.5\times 10^{-5}$ (10 dB), confirming that the deviation from the original signal is minor. PSNR and PRD results further highlight this trend: in clean conditions, PSNR median values reach 41.9 dB with PRD as low as $1.5\%$, showing excellent morphological preservation. Under noisy conditions, PSNR decreases (to 20–25 dB) and PRD rises (up to $19\%$), reflecting noise dependence, but still within an acceptable range for ECG analysis.

**Table 10:** Acquired results from Empirical Mode Decomposition filtering experiment.

Metrices	EMD(signal)				EMD(signal + 10dB AWGN)				EMD(signal + 15dB AWGN)			
	min	max	mean	median	min	max	mean	median	min	max	mean	median
**MSE**	$2.183\times 10^{-13}$	$7.598\times 10^{-06}$	$6.544\times 10^{-08}$	$3.974\times 10^{-12}$	$4.103\times 10^{-11}$	$7.718\times 10^{-06}$	$5.350\times 10^{-08}$	$1.210\times 10^{-09}$	$1.363\times 10^{-11}$	$7.612\times 10^{-06}$	$4.958\times 10^{-08}$	$4.058\times 10^{-10}$
**RMSE**	$4.672\times 10^{-07}$	$2.757\times 10^{-03}$	$8.429\times 10^{-05}$	$1.994\times 10^{-06}$	$6.406\times 10^{-06}$	$2.778\times 10^{-03}$	$9.331\times 10^{-05}$	$3.479\times 10^{-05}$	$3.692\times 10^{-06}$	$2.759\times 10^{-03}$	$7.796\times 10^{-05}$	$2.014\times 10^{-05}$
**PSNR**	-53.869	68.385	36.518	41.917	-44.149	35.986	17.646	20.292	-40.174	41.774	21.763	24.821
**PRD (%)**	0.045	195.673	17.892	1.495	8.186	157.596	26.514	19.226	4.753	112.048	19.713	11.170
**Correlation**	$7.587\times 10^{-03}$	1.000	0.883	1.000	-0.017	0.995	0.857	0.940	$2.318\times 10^{-03}$	0.998	0.902	0.978
**SNR (dB)**	-5.831	66.897	32.064	36.506	-3.951	21.739	13.191	14.322	-0.988	26.461	17.308	19.039
**Kurtosis**	-1.667	290.061	7.208	5.575	-1.621	200.813	5.629	3.425	-1.713	265.127	6.573	4.465
**Skewness**	-5.345	6.021	0.254	0.121	-6.232	10.353	0.203	0.086	-8.202	12.997	0.240	0.135
**IQR**	$4.095\times 10^{-06}$	$7.473\times 10^{-04}$	$9.230\times 10^{-05}$	$6.710\times 10^{-05}$	$1.612\times 10^{-05}$	$1.022\times 10^{-03}$	$1.300\times 10^{-04}$	$1.029\times 10^{-04}$	$1.367\times 10^{-05}$	$9.515\times 10^{-04}$	$1.140\times 10^{-04}$	$8.884\times 10^{-05}$
**Std Dev**	$1.778\times 10^{-05}$	$7.249\times 10^{-04}$	$9.727\times 10^{-05}$	$8.856\times 10^{-05}$	$1.954\times 10^{-05}$	$9.732\times 10^{-04}$	$1.219\times 10^{-04}$	$1.111\times 10^{-04}$	$1.834\times 10^{-05}$	$9.582\times 10^{-04}$	$1.119\times 10^{-04}$	$1.028\times 10^{-04}$

The very high maximum PRD values observed (up to 195.6% at 0 dB, 157.6% at 10 dB, and 112.0% at 15 dB) indicate occasional extreme deviations between the reconstructed and original ECG signals. Such cases arise when the Empirical Mode Decomposition fails to properly separate noise and oscillatory components, a phenomenon known as mode mixing. In these instances, relevant cardiac features (e.g., QRS complexes or P waves) may be partially lost or split across multiple IMFs, resulting in reconstruction errors whose energy exceeds that of the original signal (PRD > 100%). This effect is most pronounced under heavy noise conditions, where the algorithm struggles to distinguish between noise-dominated and signal-dominated modes. As the SNR improves (10 dB and 15 dB), the maximum PRD values decrease, though outlier decompositions remain. Importantly, these maxima represent only isolated pathological cases rather than the typical performance. The mean and median PRD values remain much lower (below 27% even at 10 dB), showing that in the majority of trials, EMD preserves the essential morphology of the ECG signal. Overall, the wide range of PRD values highlights the variability of EMD-based filtering: while it is capable of producing highly accurate reconstructions (median PRD $\approx 1.5\%$ at 0 dB), its sensitivity to noise and mode mixing occasionally leads to substantial reconstruction failures. This suggests that EMD is effective for denoising in most cases, but should be combined with additional safeguards (e.g., post-processing or noise-assisted variants such as EEMD) to mitigate extreme outlier behavior.

Correlation and SNR metrics corroborate these findings. Median correlation coefficients remain strong across all conditions, ranging from 0.94 (10 dB) to 1.0 (0 dB), indicating that the temporal alignment and waveform structure are well preserved. Similarly, SNR improves substantially after filtering, with medians of 36.5 dB in original signals and 14–19 dB in noisy conditions, highlighting the effectiveness of EMD in suppressing noise without excessively distorting the underlying morphology. Statistical measures reveal that the filtered signals retain realistic variability, though with some artifacts. Kurtosis remains elevated relative to Gaussian distributions, with medians of 3.4–5.6, suggesting the presence of sharp peaks introduced or preserved by decomposition. Skewness remains close to zero on average, reflecting approximate symmetry in the reconstructed signals, though extreme outliers are observed. Variability measures such as IQR and standard deviation remain stable across noise levels, with median IQR values ranging from $6.7\times 10^{-5}$ to $1.0\times 10^{-4}$ and standard deviations ranging from $8.9\times 10^{-5}$ to $1.1\times 10^{-4}$, confirming that the intrinsic dispersion of the ECG waveform is preserved.

**Fig. 13:**
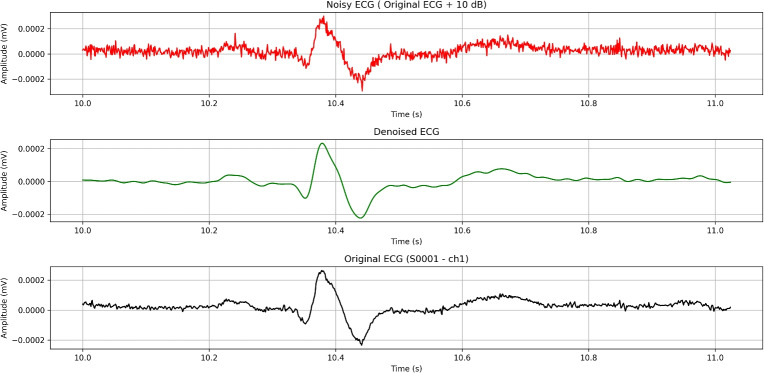
Effect of Empirical Mode Decomposition filtering on an ECG signal (~1-second ECG segment).

Overall, the EMD filter provides a good compromise between noise reduction and signal preservation (Fig. 13). While high noise levels reduce PSNR and correlation, the method consistently maintains acceptable morphological fidelity and statistical integrity, making it suitable for ECG preprocessing where adaptive decomposition is advantageous.

## Empirical wavelet transform filtering

The Empirical Wavelet Transform (EWT) is an adaptive time-frequency analysis method that constructs wavelet filter banks directly from the Fourier spectrum of a given signal. Unlike traditional wavelet transforms that rely on predefined mother wavelets, EWT adaptively partitions the Fourier spectrum into empirically determined subbands, enabling more flexible representation of non-stationary signals. The decomposition boundaries are estimated using spectral segmentation methods such as local maxima, local minima, or scale-space analysis, ensuring that each band captures relevant oscillatory content. Among these, the method based on identifying both local maxima and minima in the smoothed Fourier spectrum has demonstrated high effectiveness in detecting meaningful transitions in biomedical signals and is particularly suitable for ECG applications. This adaptive nature makes EWT highly appropriate for ECG, which exhibit non-stationary frequency characteristics due to complex physiological events.

In denoising tasks, EWT enables the separation of high-frequency noise components from diagnostically significant low- and mid-frequency bands associated with the P wave, QRS complex, and T wave. Noise suppression is typically achieved through shrinkage techniques applied to selected wavelet components, with soft thresholding offering smoother results and better morphological preservation. Compared to EMD, EWT benefits from a more stable and mathematically rigorous framework, reducing artifacts such as mode mixing and redundancy. For optimal ECG denoising, it is advisable to decompose the signal into a moderate number of frequency bands–typically between three and seven–to achieve a balance between resolution and computational stability. Further enhancement is obtained by selectively reconstructing only those subbands that represent meaningful cardiac information while excluding the lowest bands (which may contain baseline wander) and the highest bands (often dominated by high-frequency noise). Finally, cross-validation over multiple EWT configurations is recommended to identify the most suitable combination of decomposition depth, retained frequency bands, thresholding strategy, and boundary segmentation method, ensuring robust noise reduction while preserving clinical interpretability.

The parameter tuning was carried out by evaluating all possible combinations of the selected parameters. The number of modes was fixed at 4, while the thresholding strategy was varied between ’soft’ and ’hard’. The threshold value was explored across ten different settings (0, 1.5, 2, 2.5, 5, 7.5, 10, 12.5, 15, 25), and the boundary detection method was tested using both ’locmax’ and ’locmaxmin’. This exhaustive grid search resulted in a total of 40 unique parameter configurations, ensuring comprehensive coverage of the parameter space. The optimal configuration was determined to be four modes, retaining bands 3–4, soft thresholding with a threshold value of 0.075, and the ‘locmaxmin’ boundary method, which achieved the best filtering performance.

**Table 11:** Acquired results from Empirical Wavelet Transform filtering experiment.

Metrices	EWT(signal)				EWT(signal + 10dB AWGN)				EWT(signal + 15dB AWGN)			
	min	max	mean	median	min	max	mean	median	min	max	mean	median
**MSE**	$6.056\times 10^{-11}$	$6.430\times 10^{-07}$	$7.646\times 10^{-09}$	$3.362\times 10^{-09}$	$7.017\times 10^{-11}$	$6.810\times 10^{-07}$	$9.490\times 10^{-09}$	$4.208\times 10^{-09}$	$6.351\times 10^{-11}$	$6.452\times 10^{-07}$	$8.152\times 10^{-09}$	$3.718\times 10^{-09}$
**RMSE**	$7.782\times 10^{-06}$	$8.019\times 10^{-04}$	$7.063\times 10^{-05}$	$5.799\times 10^{-05}$	$8.377\times 10^{-06}$	$8.252\times 10^{-04}$	$7.901\times 10^{-05}$	$6.487\times 10^{-05}$	$7.969\times 10^{-06}$	$8.033\times 10^{-04}$	$7.350\times 10^{-05}$	$6.098\times 10^{-05}$
**PSNR**	-38.488	34.462	15.264	17.277	-40.011	33.855	14.271	16.504	-38.627	34.354	14.868	17.029
**PRD (%)**	2.339	95.486	34.712	33.046	8.684	94.203	36.806	34.847	5.422	94.292	35.497	33.643
**Correlation**	0.596	0.999	0.963	0.962	0.220	0.995	0.938	0.949	0.587	0.997	0.954	0.956
**SNR (dB)**	0.401	32.620	10.809	9.618	0.519	21.226	9.816	9.157	0.511	25.316	10.413	9.462
**Kurtosis**	-1.724	34.781	5.016	3.071	-1.708	36.269	4.792	2.854	-1.725	36.147	4.946	3.018
**Skewness**	-4.846	5.374	0.234	0.201	-5.160	5.298	0.219	0.171	-4.843	5.315	0.229	0.186
**IQR**	$1.306\times 10^{-05}$	$2.415\times 10^{-03}$	$1.589\times 10^{-04}$	$1.125\times 10^{-04}$	$1.627\times 10^{-05}$	$2.587\times 10^{-03}$	$1.648\times 10^{-04}$	$1.180\times 10^{-04}$	$1.543\times 10^{-05}$	$2.437\times 10^{-03}$	$1.606\times 10^{-04}$	$1.144\times 10^{-04}$
**Std Dev**	$2.028\times 10^{-05}$	$2.052\times 10^{-03}$	$1.717\times 10^{-04}$	$1.430\times 10^{-04}$	$2.030\times 10^{-05}$	$2.125\times 10^{-03}$	$1.745\times 10^{-04}$	$1.456\times 10^{-04}$	$2.054\times 10^{-05}$	$2.051\times 10^{-03}$	$1.725\times 10^{-04}$	$1.439\times 10^{-04}$

**Fig. 14:**
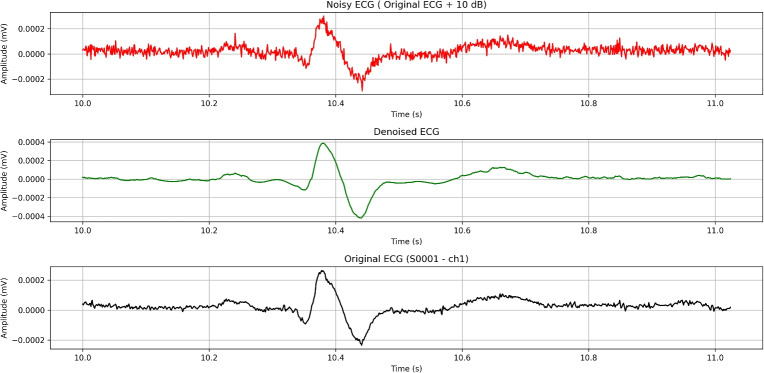
Effect of Empirical Wavelet Transform filtering on an ECG signal (~1-second ECG segment).

The EWT filter demonstrates consistent denoising performance across noise levels, as reflected in the error metrics, correlation measures, and statistical characteristics (Table 11). The MSE and RMSE values remain low across all conditions, with mean MSE values on the order of $10^{-9}$ and RMSE means around $7\!\times \!10^{-5}$, indicating that the reconstruction error is minor relative to the signal amplitude. Correspondingly, the PSNR values exhibit median levels between 16–17 dB, suggesting moderate fidelity preservation, although the minimum values highlight occasional decompositions with degraded quality. The PRD results further confirm this behavior: median PRD values remain stable (approximately 33–35%), showing that the majority of reconstructions maintain a reasonably close match to the original ECG morphology. However, maximum PRD values approaching 95% at all noise levels indicate that certain decompositions suffer from significant deviations, reflecting sensitivity of EWT to mode allocation in specific signals. Despite these extremes, the correlation coefficients remain strong, with mean values above 0.93 and medians consistently above 0.94, reinforcing that essential morphological features are generally retained. The SNR results follow a similar trend, with median values around 9–10 dB across conditions, representing a measurable improvement compared to the noisy inputs.

From a statistical perspective, the filtered signals maintain physiologically plausible distributional properties. Kurtosis values (mean $\approx$ 5) suggest the presence of sharp ECG events such as QRS complexes, while skewness remains near zero on average, indicating that the signal distributions are nearly symmetric after denoising. The IQR and standard deviation values remain consistent across noise levels, reflecting preserved variability without over-smoothing.

Overall, the EWT filter effectively reduces noise while retaining essential ECG characteristics, showing robustness across 0 dB, 10 dB (Fig. 14), and 15 dB noise conditions. Nonetheless, the presence of high PRD outliers indicates that EWT, while generally reliable, can occasionally misrepresent localized signal features, and thus benefits from careful parameter tuning or hybrid approaches in critical clinical applications.

## Stationary Wavelet Transform filtering

Finally, we will use SWT for filtering ECG data. A key consideration when dealing with SWT filtering is the careful selection of wavelet type, decomposition level, and thresholding method, as these parameters significantly influence the filter’s effectiveness. The wavelet type was varied across ten options: ’db4’, ’db5’, ’db6’, ’sym4’, ’sym5’, ’coif3’, ’coif4’, ’coif5’, ’bior3.5’, and ’rbio3.9’. The decomposition level was varied from 3 to 6, and the threshold scaling factor was explored over a range of 0.3 to 1.0 in steps of 0.1. This exhaustive search ensured that all feasible configurations were tested, providing a comprehensive exploration of the parameter space. The optimal configuration was determined to be the wavelet type ‘rbio3.9’, level 5, and a threshold scale of 0.5. These parameters and their corresponding values were carefully selected to balance noise reduction and signal accuracy, optimizing the filter’s effectiveness for the specific characteristics of the measured data.

The SWT filter was applied to ECG signals contaminated with 0, 10, and 15 dB AWGN, and its performance was evaluated using MSE, RMSE, SNR, PRD, correlation, PSNR, and statistical descriptors (Table 12). Across all noise levels, SWT achieved very low reconstruction errors, with median MSE between $9.288\times 10^{-11}$ and $2.109\times 10^{-10}$ and median RMSE from $3.499\times 10^{-06}$ to $1.452\times 10^{-05}$, confirming high-fidelity waveform recovery. Signal quality improved markedly, with SNR increasing from an original mean of 16.497 dB to 34.9 dB after SWT filtering, while median PSNR reached 40.474 dB, indicating well-preserved peak features. PRD values remained low (2.689–8.269 %), and correlation coefficients consistently exceeded 0.981, with a mean of 0.998, demonstrating near-perfect similarity to the original signals. Statistical measures (kurtosis, skewness, IQR, and standard deviation) closely matched the unfiltered signal, suggesting minimal distortion of amplitude distributions, though minor variability in skewness and kurtosis pointed to subtle distributional changes. The narrow IQR of $1.316\times 10^{-04}$ further indicated tightly clustered central amplitudes, reflecting stability and effective suppression of noise fluctuations. SWT effectively preserved both high- and low-frequency components, retaining clinically important features such as QRS complexes and P/T waves, and demonstrated superior robustness compared with conventional filtering methods, making it well suited for physiologically faithful ECG denoising under both high-noise and routine acquisition conditions.

**Table 12:** Acquired results from SWT filtering experiment.

Metrices	swt(signal)				swt(signal + 10dB AWGN)				swt(signal + 15dB AWGN)			
	min	max	mean	median	min	max	mean	median	min	max	mean	median
**MSE**	$5.324\times 10^{-13}$	$2.162\times 10^{-09}$	$2.371\times 10^{-11}$	$1.225\times 10^{-11}$	$8.486\times 10^{-12}$	$1.810\times 10^{-08}$	$5.911\times 10^{-10}$	$2.109\times 10^{-10}$	$4.541\times 10^{-12}$	$8.477\times 10^{-09}$	$2.327\times 10^{-10}$	$9.288\times 10^{-11}$
**RMSE**	$7.297\times 10^{-07}$	$4.650\times 10^{-05}$	$4.089\times 10^{-06}$	$3.499\times 10^{-06}$	$2.913\times 10^{-06}$	$1.345\times 10^{-04}$	$1.935\times 10^{-05}$	$1.452\times 10^{-05}$	$2.131\times 10^{-06}$	$9.207\times 10^{-05}$	$1.247\times 10^{-05}$	$9.638\times 10^{-06}$
**PSNR**	-17.272	62.836	39.405	40.474	-31.147	50.863	26.632	28.777	-26.557	53.088	30.182	32.362
**PRD (%)**	0.093	49.662	2.689	1.890	3.770	61.035	8.269	7.680	2.158	49.764	5.650	5.046
**Correlation**	0.855	1.000	0.998	0.999	0.438	0.999	0.981	0.991	0.669	1.000	0.992	0.996
**SNR (dB)**	6.079	60.660	34.950	34.471	4.288	28.472	22.177	22.293	6.062	33.321	25.727	25.942
**Kurtosis**	-1.765	391.162	6.965	4.565	-1.727	379.199	6.160	3.755	-1.750	377.435	6.538	4.097
**Skewness**	-6.293	10.985	0.240	0.152	-5.657	10.292	0.217	0.131	-5.711	10.730	0.228	0.143
**IQR**	$1.088\times 10^{-05}$	$2.333\times 10^{-03}$	$1.316\times 10^{-04}$	$8.591\times 10^{-05}$	$1.198\times 10^{-05}$	$2.345\times 10^{-03}$	$1.336\times 10^{-04}$	$8.851\times 10^{-05}$	$1.143\times 10^{-05}$	$2.312\times 10^{-03}$	$1.322\times 10^{-04}$	$8.682\times 10^{-05}$
**Std Dev**	$1.743\times 10^{-05}$	$1.468\times 10^{-03}$	$1.240\times 10^{-04}$	$1.021\times 10^{-04}$	$1.747\times 10^{-05}$	$1.470\times 10^{-03}$	$1.238\times 10^{-04}$	$1.017\times 10^{-04}$	$1.740\times 10^{-05}$	$1.469\times 10^{-03}$	$1.235\times 10^{-04}$	$1.015\times 10^{-04}$

**Fig. 15:**
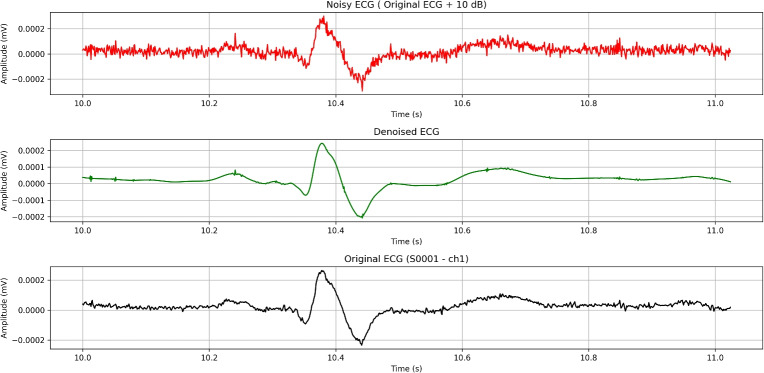
Effect of Stationary Wavelet Transform filtering on an ECG signal (~1-second ECG segment).

In summary, the SWT filtering method proves highly effective in maintaining signal integrity, as evidenced by the high SNR, PSNR, and correlation coefficient values, coupled with low MSE and RMSE metrics. However, the Kurtosis and Skewness suggest that while the filtering is precise, its impact on noise reduction may be subtle, particularly when starting with relatively clean signals (Fig. 15). The filtered signal’s distribution is therefore nearly symmetric, slightly flatter than normal, lacks heavy tails or extreme outliers. This often suggests a well-filtered signal (like a clean ECG after denoising), stable data, with minimal deviation or irregular spikes.

## Runtime characteristics of filtering methods

The simplest method, the moving average filter, operates in linear time and is highly efficient for smoothing short-term noise in ECG recordings. Closely related is the SGS filter, which also scales linearly but provides better preservation of morphological features such as the P wave, QRS complex, and T wave. The Kalman filter, although also operating in linear time, introduces a slightly larger computational overhead because of its recursive state-update equations. Despite this, it remains well-suited for real-time ECG applications where accurate estimation of underlying cardiac dynamics is required. The next group of filters consists of classical digital IIR or FIR designs, namely the notch filter, high-pass filter, and Chebyshev Type II filter.

**Fig. 16:**
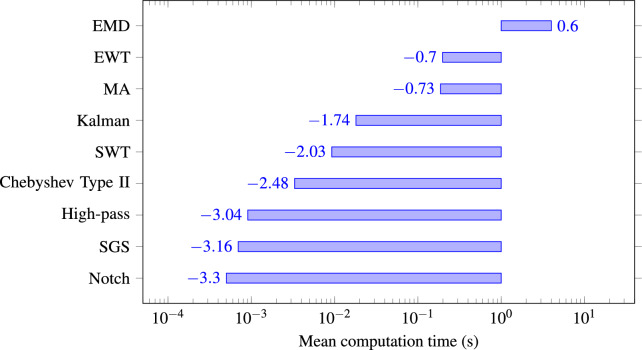
Measured mean computation times of ECG denoising filters. Results are shown on a logarithmic scale to highlight differences between fast linear filters (e.g., SGS, notch, high-pass) and adaptive methods (e.g., EWT, EMD) which are considerably slower.

These filters typically exhibit a complexity proportional to the product of the signal length and the filter order. Because the filter order *d* is usually small (e.g., 2–10), their practical runtime is still close to linear, making them efficient choices for baseline wander or PLI removal. The notch filter is particularly effective at attenuating a very narrow frequency band, such as 50 Hz or 60 Hz, with minimal distortion to the remaining ECG signal. The high-pass filter is commonly applied to remove baseline wander, which results from respiration and electrode drift, while maintaining low-frequency ECG components. The Chebyshev Type II filter is advantageous when sharp stopband attenuation is needed, although it introduces ripples in the stopband as a trade-off. Moving beyond classical filters, transform-based methods such as the SWT are more computationally demanding. SWT avoids downsampling, which improves feature preservation, but this redundancy increases its complexity. The EWT is even more adaptive, as it partitions the Fourier spectrum into empirically determined bands. Its complexity arises from the use of FFT operations combined with mode-specific filtering. While EWT is more expensive than SWT, it can provide better adaptability for nonstationary signals like ECG. At the highest end of the computational spectrum lies EMD. This iterative and nonlinear technique requires repeated envelope interpolations and sifting operations. As a result, EMD is substantially slower than the other approaches, yet it offers unmatched adaptability for complex biomedical signals. Overall, the table highlights a clear trade-off: simpler filters offer speed and efficiency, while adaptive methods like EWT and EMD provide flexibility and higher signal fidelity at a significant computational cost.

In our experiments (benchmarked on Intel(R) Xeon(R) CPU E5-2670 v3 @ 2.30GHz) the notch filter was the fastest (Fig. 16), with a mean execution time of only $0.000503$ seconds, followed closely by the SGS filter at 0.000695 seconds and the high-pass filter at $0.000908$ seconds. These three filters therefore provide near-instantaneous processing and are highly suitable for real-time ECG denoising. The Chebyshev Type II filter also performed very efficiently, with a mean time of $0.003312$ seconds, offering sharp stopband attenuation at low cost. The SWT required more computation, with a mean time of 0.009262 seconds, reflecting its redundancy from avoiding downsampling. The Kalman filter, although theoretically linear, showed a mean execution time of 0.018066 seconds due to its recursive state-update steps, making it somewhat slower but still feasible for real-time applications. The MA filter was observed to be slower than expected, with a mean time of 0.187352 seconds, likely due to implementation details, yet it remains lightweight compared to more advanced transforms. The EWT exhibited a mean of 0.198301 seconds, clearly indicating its higher cost associated with FFT operations and adaptive spectral partitioning. Finally, EMD were the slowest, with mean times of 4.006334 seconds, although their maximum values exceeded 30 seconds in certain cases. These results confirm the theoretical expectations: simple linear filters such as SGS, notch, and high-pass are the most efficient, while adaptive methods like EWT and EMD are considerably slower. The median times reflect the same trend, with EWT and EMD having the largest variability across runs. Overall, the measurements highlight a clear trade-off: faster filters are ideal for embedded or real-time monitoring, whereas computationally heavier methods are more suitable for offline analysis where adaptability and accuracy are prioritized.

For real-time ECG processing, lightweight filters such as the moving average, SGS, Kalman, notch, and high-pass filters are most appropriate. These methods operate in linear time with very low computational overhead, making them suitable for deployment in wearable devices, bedside monitors, and mobile health applications. The moving average filter provides basic smoothing, while the SGS filter preserves morphological details, which is essential when detecting subtle abnormalities in ECG waveforms. The Kalman filter is particularly valuable in real-time contexts because it not only denoises the signal but also continuously estimates the underlying cardiac state in a recursive manner. Notch filters and high-pass filters are also indispensable in real-time systems because they effectively remove PLI and baseline wander with minimal delay. On the other hand, offline analysis scenarios can take advantage of more computationally expensive approaches such as SWT, EWT, and EMD. SWT is well-suited for detailed post-processing because of its redundancy and strong denoising ability across multiple frequency bands. EWT provides adaptive spectral decomposition that captures nonstationary behavior, making it ideal for research into arrhythmias or transient ECG events. EMD, while computationally slow, offers unmatched flexibility for decomposing highly nonlinear and nonstationary signals, which makes it a powerful offline tool for research studies, diagnostic model development, and comparative denoising experiments. In summary, real-time systems prioritize efficiency and responsiveness, whereas offline research analysis emphasizes adaptability and accuracy, leading to different filter choices depending on the application.

## Achieved results from conducted experiments

We have carefully examined the acquired results. We observed the evaluation metrics and considered every aspect of them in detail. In addition, we analyzed the computation time of the applied filtering methods to assess their practical feasibility for deployment in wearable devices. The experimental results clearly demonstrated that SWT filtering achieved very good overall performance. Here are the summarized reasons supporting this conclusion.

The choice of the SWT filter for processing ECG signals, particularly for the PTB diagnostic database, is done by its performance across a range of critical signal quality metrics. The ECG signals are somewhat complex, containing both low- frequency components like P-waves, T-waves, and high-frequency components such as QRS complexes. The SWT filter’s ability to denoise while preserving these essential signal characteristics makes it an optimal choice.

The comparative analysis (Table 13) of filtering techniques highlights clear differences in robustness and denoising quality. Classical approaches such as the Chebyshev Type II and high-pass filters show consistently poor performance, with PRD values near 90 % and correlations below 0.75, indicating significant distortion of ECG morphology. Empirical Mode Decomposition performs well on the clean signal, achieving low RMSE and high correlation, but its effectiveness collapses under 10 dB AWGN, where PRD rises to $\approx 19$ % and correlation drops to 0.587, reflecting a lack of robustness. The EWT yields high correlation values above 0.93, but its consistently elevated PRD ($\approx 33$ %) suggests weak amplitude fidelity and limited noise suppression. The Kalman filter achieves balanced results with correlations around 0.92 and PRD near 20 %, displaying stability across noise levels but falling short of the top-performing methods. The moving average filter follows a similar trend, with slightly worse performance (PRD 24–25 %, correlation near 0.88), reflecting oversmoothing effects.

**Table 13:** Comparison of filtering techniques based on mean of RMSE, median of PSNR, median of PRD (%) and mean of correlation.

filter	filter(signal)				filter(signal + 10dB AWGN)				filter(signal + 15dB AWGN)			
	RMSE	PSNR	PRD (%)	Correlation	RMSE	PSNR	PRD (%)	Correlation	RMSE	PSNR	PRD (%)	Correlation
**Chebyshev Type II**	$2.610\times 10^{-4}$	8.225	91.083	0.700	$2.619\times 10^{-4}$	8.181	91.385	0.674	$2.613\times 10^{-4}$	8.203	91.210	0.689
**EMD**	$8.429\times 10^{-5}$	32.064	1.495	0.883	$9.331\times 10^{-5}$	13.191	19.226	0.587	$7.796\times 10^{-5}$	17.308	11.170	0.902
**EWT**	$7.063\times 10^{-5}$	10.809	33.046	0.963	$7.901\times 10^{-5}$	9.816	34.847	0.938	$7.350\times 10^{-5}$	10.413	33.643	0.954
**High-pass**	$2.566\times 10^{-4}$	8.405	89.688	0.751	$2.734\times 10^{-4}$	7.635	95.583	0.586	$2.622\times 10^{-4}$	8.138	91.849	0.663
**Kalman**	$3.741\times 10^{-5}$	21.790	19.833	0.929	$4.462\times 10^{-5}$	20.802	21.437	0.910	$4.025\times 10^{-5}$	21.401	20.352	0.922
**MA**	$4.586\times 10^{-5}$	19.890	24.181	0.890	$5.226\times 10^{-5}$	19.159	25.339	0.872	$4.831\times 10^{-5}$	19.644	24.584	0.884
**Notch**	$2.003\times 10^{-6}$	47.796	0.841	0.999	$8.405\times 10^{-5}$	16.628	30.073	0.825	$4.733\times 10^{-5}$	21.604	16.952	0.919
**SGS**	$8.865\times 10^{-6}$	33.876	4.413	0.994	$2.431\times 10^{-5}$	26.979	9.282	0.972	$1.612\times 10^{-5}$	30.221	6.483	0.987
**SWT**	$4.089\times 10^{-6}$	41.917	1.890	0.998	$1.935\times 10^{-5}$	20.292	7.680	0.981	$1.247\times 10^{-5}$	24.821	5.046	0.992

The notch filter excels on the clean signal (RMSE $2.003\times 10^{-6}$, PSNR 47.796 dB, correlation 0.999), but its performance deteriorates substantially in broadband noise (PRD $\approx 30$ % at 10 dB, correlation 0.825), underscoring its specialization for narrow-band interference rather than general ECG denoising. The SGS demonstrates strong performance, with low RMSE, high PSNR in the 27–34 dB range, PRD below 10 % under noise, and correlations above 0.97. The SWT achieving the lowest RMSE, the highest PSNR (41.9 dB on clean signals and remaining above 20 dB with noise), the lowest PRD (1.9–7.7 %), and extremely high correlations (0.98–0.998), while maintaining stability under noise. Considering the kurtosis and skewness metrics (Table 12), the SWT filter maintains consistent values that reflect its ability to preserve the statistical properties of the original signal. These metrics further indicate that the SWT filter does not introduce any significant distortion to the signal’s distribution, maintaining the natural characteristics of the ECG data. This confirms SWT as the most reliable and accurate approach for ECG denoising.

**Fig. 17:**
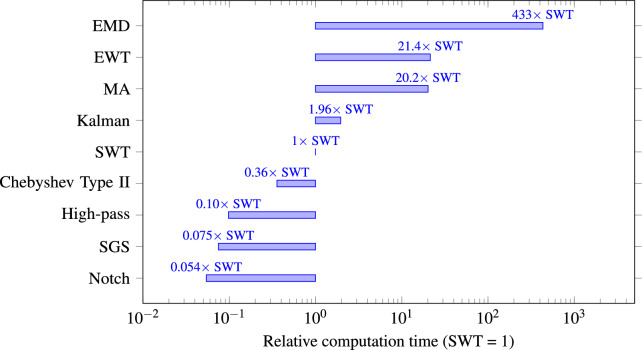
Relative computation times of ECG denoising filters with the SWT set as baseline. Values less than $1\times$ SWT indicate faster execution, while values greater than $1\times$ SWT denote slower methods.

With regard to computational time, the SWT exhibits an intermediate runtime compared to other filtering methods. The SWT showed a higher computation time (Fig. 17) than that of the classical linear-time filters such as SGS, notch, high-pass, and Chebyshev II, but still well below the costs of adaptive approaches like EWT and EMD. This intermediate runtime reflects the redundant nature of SWT: since no downsampling is performed, each decomposition level requires processing the full signal length. As a result, SWT has a theoretical complexity of $O(N \cdot L)$, where L is the number of decomposition levels. In practice, this redundancy improves feature preservation across frequency scales, which is highly valuable for ECG denoising tasks where the preservation of P waves, QRS complexes, and T waves is essential. Therefore, SWT represents a balance between computational efficiency and denoising quality, being slower than traditional filters but significantly faster than fully adaptive decomposition methods, which makes this method suitable for implementation on lower-performance CPUs, such as those embedded in wearable devices.

## Conclusion

This study addresses advancements in ECG signal filtering to improve the accuracy of cardiovascular disease detection systems. Among the evaluated methods, the SWT emerged as the most effective technique for ECG signal denoising with respect to computational efficiency and denoising performance. The SWT demonstrated superior capability in attenuating noise while preserving diagnostically relevant features of the signal, with computational demands sufficiently low to enable its integration into wearable devices intended for continuous patient monitoring.

This study highlights the importance of robust preprocessing techniques in ensuring the reliability of ECG signal processing, contributing to advancements in the field of cardiovascular monitoring. Future work could explore the application of SWT in combination with other biosignals to further improve signal quality and diagnostic accuracy.

## Data Availability

The dataset analysed during the current study is available at https://www.kaggle.com/datasets/physionet/ptb-diagnostic-ecg-database.
